# Orienting to fear under transient focal disruption of the human amygdala

**DOI:** 10.1093/brain/awac032

**Published:** 2022-02-01

**Authors:** Ashwani Jha, Beate Diehl, Bryan Strange, Anna Miserocchi, Fahmida Chowdhury, Andrew W McEvoy, Parashkev Nachev

**Affiliations:** UCL Queen Square Institute of Neurology, London, UK; UCL Queen Square Institute of Neurology, London, UK; CTB-UPM and Department of Neuroimaging, Reina Sofia Centre for Alzheimer's Research, Madrid, Spain; UCL Queen Square Institute of Neurology, London, UK; UCL Queen Square Institute of Neurology, London, UK; UCL Queen Square Institute of Neurology, London, UK; UCL Queen Square Institute of Neurology, London, UK

**Keywords:** amygdala, fear, electrical stimulation, drift-diffusion, gamma oscillations

## Abstract

Responding to threat is under strong survival pressure, promoting the evolution of systems highly optimized for the task. Though the amygdala is implicated in ‘detecting’ threat, its role in the action that immediately follows—‘orienting’—remains unclear. Critical to mounting a targeted response, such early action requires speed, accuracy, and resilience optimally achieved through conserved, parsimonious, dedicated systems, insured against neural loss by a parallelized functional organization. These characteristics tend to conceal the underlying substrate not only from correlative methods but also from focal disruption over time scales long enough for compensatory adaptation to take place.

In a study of six patients with intracranial electrodes temporarily implanted for the clinical evaluation of focal epilepsy, we investigated gaze orienting to fear during focal, transient, unilateral direct electrical disruption of the amygdala. We showed that the amygdala is necessary for rapid gaze shifts towards faces presented in the contralateral hemifield regardless of their emotional expression, establishing its functional lateralization. Behaviourally dissociating the location of presented fear from the direction of the response, we implicated the amygdala not only in detecting contralateral faces, but also in automatically orienting specifically towards fearful ones. This salience-specific role was demonstrated within a drift-diffusion model of action to manifest as an orientation bias towards the location of potential threat. Pixel-wise analysis of target facial morphology revealed scleral exposure as its primary driver, and induced gamma oscillations—obtained from intracranial local field potentials—as its time-locked electrophysiological correlate.

The amygdala is here reconceptualized as a functionally lateralized instrument of early action, reconciling previous conflicting accounts confined to detection, and revealing a neural organisation analogous to the superior colliculus, with which it is phylogenetically kin. Greater clarity on its role has the potential to guide therapeutic resection, still frequently complicated by impairments of cognition and behaviour related to threat, and inform novel focal stimulation techniques for the management of neuropsychiatric conditions.

## Teaser

Jha *et al.* use a novel oculomotor task during transient unilateral disruption of the human amygdala to establish the functional lateralisation of the amygdala to the contralateral hemifield, and its dual role: detection of all faces and automatic orienting to fearful ones.

## Introduction

Gaze orientation is typically the first act to follow visual perception. In circumstances of environmental threat, orienting must be both fast and spatially accurate, for further, focused perception depends on it.^1^ Joint optimization of speed and accuracy is hypothetically best achieved by a simple, dedicated system that exploits the narrow range of stimuli of immediate interest—threatening objects—and the economy of the information initially required—spatial location. Such a dedicated system requires a ‘sensory’ component tailored to the detection of the target class, and a ‘motor’ component optimized for maximally rapid shifts of gaze, both operating in advance of—and in parallel with—the more complex neural mechanisms able to take advantage of the high-fidelity visual information foveating a target provides. It is a putative architecture with an established precedent in the superior colliculus-centred network that facilitates fast, reflexive orienting to low-level visual cues in parallel with cortical processing. It also facilitates a graceful evolution in perceptual flexibility, retaining established systems with survival-critical performance while novel, more sophisticated mechanisms emerge.

A phylogenetically old, amygdala-centred pathway is the most compelling candidate for such a system in the human brain.^2–5^ Correlative evidence from multiple experimental modalities has consistently shown neural sensitivity to emotionally salient visual stimuli.^6–9^ But distinguishing between serial and parallel pathway configurations is difficult with correlative data, for identical patterns of neural activation will typically be predicted in each case.

Disruptive methods have greater power to illuminate the underlying causal organization. They are, however, difficult to apply to so small and inaccessible a region. Long-standing bilateral amygdala damage impairs spontaneous recognition of fear in a face,^10,11^ but not when verbally-guided attention recruits parallel, presumably neocortical, mechanisms.^12^ Though this seminal finding suggests a dual pathway, its generality is complicated by the chronicity of the damage in a single, rare patient, where elaborate adaptive changes, both neural and behavioural, have had time to develop.

The rich afferent connectivity of the amygdala, placing it in receipt of fast visual information via multiple parallel cortical and subcortical pathways,^3,4,13–16^ may be thought to favour a dominant ‘sensory’ role in fear recognition—whether within a dual or a unitary pathway. The behavioural findings, however, are conflicting. Though rapid detection of faces was impaired in one patient,^17^ non-conscious detection of fearful faces was unaffected in another,^18^ and five patients with lesions restricted to the basolateral subnuclei of the amygdala were hypersensitive to detecting fear.^19^

Nor is it clear how the amygdala contributes to a spatially specific ‘oculomotor’ response. Microstructure of the human stria terminalis—which carries amygdala efferents—correlates with orientation to threat but without lateralisation.^20^ Amygdala neurons in non-human primates have been shown to encode a contralaterally biased hemifield expectation of reward or punishment following presentation of low-level visual cues.^21,22^ But saccadic responses were here dissociated from the cue itself, obscuring the relevance for action of this ‘perceptual’ facility. Indeed, under free-viewing conditions, unilateral amygdala lesions in humans impair ‘ipsilateral’ use of emotionally-salient information only subtly.^23^ This raises the possibilities that amygdala encoding of hemifield salience may require operant conditioning, may be transmitted from nearby structures, or may be less relevant in humans where—unlike non-human primates^24^—the amygdalae are far more tightly interconnected.^21^ Human amygdala lesion-deficit models may also be imprecise, confounded by chronic adaptation, collateral pathological damage, or inter-subject functional heterogeneity.

To resolve these long-standing questions, here we employ focal, transient, direct electrical disruption of the human amygdala, revealing a comprehensive picture of its role in behaviour under threat. In sharp distinction to previous human lesion studies, our cohort of six patients implanted with intracranial EEG (iEEG) electrodes for clinical investigation of focal epilepsy uniquely enables within-subject unilateral manipulation of the neural substrate. Combining disruption with a novel oculomotor behavioural paradigm that dissociates sensory and motor hemifields, we illuminate the cardinal role of the amygdala in rapid orienting towards emotionally salient objects in the contralateral hemifield and reconcile conflicts in the extant literature in favour of a dual-pathway organization of the neural substrate supporting the response to environmental threat.

## Materials and methods

### Participants

All participants had medication-resistant focal epilepsy and were being assessed for epilepsy surgery at the Sir Jules Thorn Telemetry Unit, National Hospital for Neurology and Neurosurgery, London, UK. Pre-surgical assessments seek to identify the neuroanatomical region critical to seizure initiation—the resection target—and nearby behaviourally critical regions to be surgically spared. An array of non-invasive investigations—including structural and functional imaging, surface EEG and a neuropsychological and neuropsychiatric evaluation—is in selected patients followed by invasive temporary surgical implantation of iEEG electrodes through which focal electrophysiological sampling and modulation of localized neural substrates can be conducted. The electrode implantation scheme differs across patients owing to individual tailoring to specific candidate areas. Patients are monitored with continuous iEEG and video monitoring, for (typically) 1–2 weeks until sufficient information is acquired. IEEG electrodes are used for both recording local field potentials and delivering focal electrical stimulation through brief 2–5 s pulses of 50 Hz current (applied between two adjacent electrodes, i.e. bipolar, biphasic pulse). Such disruption is routinely used to transiently and focally disrupt brain activity during cognitive and behavioural tasks. The observation of contemporaneously disrupted behaviour is taken as confirmation that a region is critical for that behaviour and remains a Gold Standard method of mapping brain function in individuals.

We recruited six participants where the clinically indicated implantation sites included either amygdala (see Supplementary Table 1 for demographic details). We invited them to undertake the behavioural tasks described below both during iEEG recording (control block) and during disruption of the amygdala (disruption block), in a within-subject block-design where order was counterbalanced across participants. This work has received ethical approval from the Health Research Authority and the local research ethics committee (ref: 16/LO/0618).

### Behaviour

#### Tasks

To disentangle the sensory and motor components of rapid orienting guided by emotional salience, we designed a novel two-alternative forced choice saccadic task that mapped the parameters of the response—latency and choice—to the features determining the comparative emotional salience of pairs of face images—the emotional expression and its local morphological characteristics. Unlike tasks previously deployed in this domain, ours sought to guide behaviour not by the presence or absence of fear but by the participant’s immediate, instinctual, impression of salience heedless of the expressed emotion. This is because we are interested in mechanisms underlying orientation in advance of the conscious perception of the target’s emotional label that would be obscured if the instruction presupposed identifying the fearful image first. Such instinctual, acategorial, salience-driven orienting has previously been shown to be behaviourally dissociable from the deliberate, categorial, identity-driven kind.^25,26^ Second, we dissociated yielding to such instinctually defined salience from opposing it in favour of the alternative response. Though counter-responding is familiar from countermanding and anti-saccade tasks,^27,28^ here—unlike there—the definition of a counter-target is the participant’s intuitively perceived inclination, not any objectively prescribed characteristic of the target. This manipulation allowed us to spatially disentangle sensory and motor components while maintaining a focus on the immediate, intuitive aspects of the behaviour. A broadly similar task has been used to dissociate variable luminance target detection and reaching following superior colliculus disruption in non-human primates.^29^

**Figure 1:**
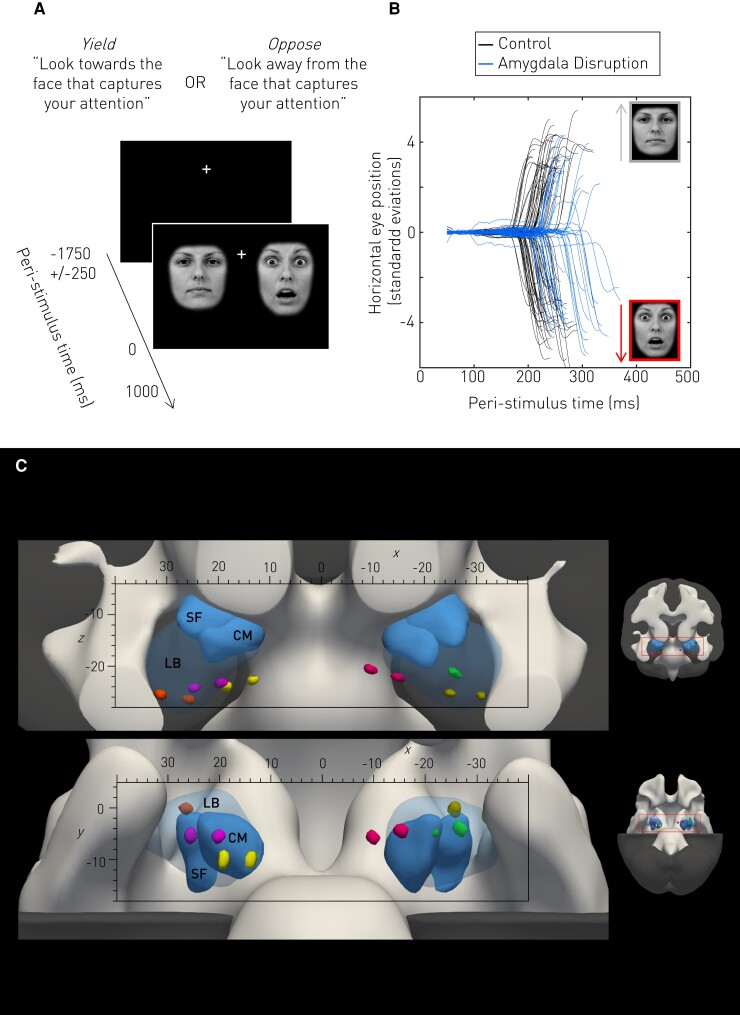
**Faces task.** During each trial, invited fixation of a central cross (**A**) was succeeded following a jittered delay by two horizontally-arranged images of the same actor wearing a neutral and fearful facial expression, the actor and fearful face side randomized across trials. Instructions were varied in a block design. In the Yield condition, participants were instructed to look towards the face that captured their attention first. In the Oppose condition, participants were asked to look away from the face that captured their attention, and towards the other face instead. The task was repeated during disruption of the amygdala, and orientation choice (fearful face or neutral face) and latency were acquired with 1 kHz eye-tracking. (**B**) The horizontal eye position of an example participant in standard deviations, against peri-stimulus time (Yield condition only). Saccade directions and onsets can be clearly identified, demonstrating increased saccadic latency with amygdala disruption. (**C**) All electrode contact locations were predominantly in the latero-basal subnuclei of the amygdala for all participants. The preoperative MR and postoperative CT images of each participant were rigidly registered to each other, and then non-linearly registered into a common group (MNI) space. Electrode contacts, hyperdense on CT, are visualized here as 3D spheroidal surfaces. The stimulating bipolar contacts for each of the six participants are shown as a coloured pair. The mean group grey and white matter images are presented in register to the electrode contacts as MR derived isointense surfaces which have been cropped to allow direct visualization of the amygdala. Amygdala subnuclei are represented as blue isoprobabilistic surfaces derived from the SPM Anatomy toolbox.^30,31^ The latero-basal (LB) subnuclei are semi-transparent, whilst the superficial (SF) and centeromedial (CM) nuclei are opaque. The main panels show a magnified sagittal (*top*) and axial (*bottom*) view of the combined image, with the corresponding axes in MNI space (in mm). The image *insets* to the *right* show the orientation of each main panel view, and the magnified area (red rectangle).

This novel behavioural ‘Faces task’ is schematized in Fig. 1. A central fixation cross of 1750 ms duration (±250 ms random jitter to minimize anticipation) was followed by a 1000 Hz auditory beep coincident with the simultaneous presentation of two human face images on either side. Each face was centred at 5.3° horizontal eccentricity, well outside the 1–3° foveal range.^32,33^ Each pair of faces consisted of a neutral and fearful expression performed by the same actor, randomly selected without replacement from a pool of 89 actors (Supplementary material). In light of the current debate over the word ‘fear’ in neuroscience research^34^ here we use the term ‘fearful’ to describe subjective ratings of normed stimuli and make no claims about any emotion thereby evoked. Face presentation lasted 1000 ms after which only the fixation cross remained on screen until the next trial. The laterality of the neutral and fearful face was randomly varied between trials to avoid anticipation.

Two main conditions were implemented in blocked fashion. In ‘Yield’ blocks, participants were instructed to focus on the fixation cross at the start of each trial and then to quickly look at the face that first captured their attention, regardless of its emotional salience. In the ‘Oppose’ condition, participants were instructed instead to look away from the face that first captured their attention, towards the other face.

In a low-level control ‘Crosses task’ executed in independent sessions, the face stimuli were replaced by bright and dark crosses leaving all other parameters the same. This addressed the question of face specificity and was performed with the Yield condition only^35^ (Supplementary material).

Both tasks were performed in blocks of 50 trials with a minute break between each block. Each block could be performed without disruption, or with periods of stimulation-induced disruption described in detail below. After one practice block, each participant performed two undisrupted blocks of each control condition and one to two disrupted blocks of each main condition, in an order counterbalanced across participants.

Fewer disruption blocks were acquired owing to clinical limits on the number of permissible pulses. Not all participants completed all conditions: everyone completed the yield condition of the Faces task, three participants additionally performed the oppose condition of the Faces task, whilst the other three participants performed the Yield condition of the Crosses task (Supplementary material).

Stimuli were presented on wall-mounted Sharp 49-inch HD (1920 × 1080) monitors with a refresh-rate of 60 Hz. For safety reasons, participants remained in bed during the cortical disruptions, but were comfortably sat up directly facing the screen at a distance of 3.25 m, subtending 22° of visual angle in the diagonal plane. Stimuli were delivered using Presentation software (Neurobehavioral Systems, Inc.), running on a wall-mounted Dell^®^ Venue Pro 11 tablet running Microsoft^®^ Windows 10. Stimuli timing data were synchronously outputted to the Micromed EEG acquisition computer as a digital channel and recorded on the tablet for offline analysis.

#### Eye tracking

Eye movements were acquired using a JAZZ-Novo head-mounted direct infra-red eye-tracker and recorded for offline analysis at 1 kHz sampling rate (Ober Consulting Sp. Z o.o, Ponzan). Horizontal saccades were detected using JazzManager v3.12 software with the criteria of initial velocity ≥5°/s, amplitude ≥0.5°, preceding fixation ≥50 ms, and duration ≥20 ms. Each saccade trace was aligned to the evoking visual stimulus, and manually checked to remove trials where fixation was inadequate in the 100 ms prior to stimulus presentation, or saccadic latency implausibly low (<100 ms). Responses greater than 1000 ms were considered absent, and the trials were discarded. After artefact rejection, 1064 trials remained from the Faces task (1300 acquired, 236 rejected) and 715 trials from the Crosses task (850 acquired, 135 rejected).

### Imaging

All imaging was performed in the course of routine clinical care, determined solely by clinical need and availability. All patients had T_1_-weighted pre-implantation images (maximum strength 40 mT/m and slew rate 150 T/m/s), and post-implantation CT images conducted to ascertain electrode placement. For each patient, the T_1_ image was rigidly realigned to Montreal Neurological Institute (MNI) coordinates and the CT co-registered to it with a heavily regularized non-linear algorithm adapted to compensate for geometric distortions.^36^ Transformation parameters for a non-linear transformation to MNI space were then derived from the T_1_, and applied to both CT and T_1_ to yield a set of electrode locations in a space common to the group (further details in the Supplementary material). Placement of electrodes was within the basolateral amygdala or closely adjacent (Fig. 1C).

### Intracranial EEG

#### Electrode implantation

The implantation scheme was determined by the clinical team, dictated solely by clinical need. Structural MRI (described above) was used to plan the implantation of multiple depth electrodes (Ad-Tech Medical Instrument Corporation). Placement of electrodes was performed with a frameless stereoencephalography (SEEG) approach through individual burr-holes for each electrode.^37^ Electrode localization was confirmed via post-operative CT as described above.

#### Data acquisition

Intracranial EEG data were acquired with a standard clinical acquisition system (Micromed, S.p.A.). Multiple 64-channel Micromed SD LTM 64 Express amplifiers were used in HeadBox mode, and data underwent 16 bit analog to digital conversion at a sampling rate of 1024 Hz. At acquisition, data were referenced to a white matter contact in the hemisphere contralateral to the investigated amygdala, with surface ground at the mastoid. Data were hardware filtered with pass-band 0.17–470 Hz and stored offline for further analysis by SystemPlusEvolution software. The identity and timing of task events were synchronously recorded by the Micromed acquisition system via a custom USB link.

#### Direct amygdala disruption

Direct cortical electrical stimulation was performed between two adjacent electrode contacts (bipolar). Trains of 50 Hz, bi-phasic square wave pulses of 500 µs width were delivered by a Micromed SD LTM STIM Cortical Stimulator module with a manual trigger operated by the attending neurologist (B.D.). Current intensity was increased over separate trains from 1 mA in increments of 0.5 mA until the occurrence of a clinically obvious change, after-discharges, or 3.0 mA (6 mA peak to peak) was reached. Each train lasted no more than 5 s. In line with clinical practice, so as to detect any task-invariant behavioural effects, disruption was initially performed in the amygdala at rest so as to derive the maximum current that could be achieved without inducing visible or patient-reported motor or sensory phenomena. The effect of stimulation was subsequently evaluated as the average performance over task blocks during intermittent 2–5 s trains of disruption at this fixed current (block design). In total, each participant received a mean of 23 amygdala disruptions (range 17–34) during task blocks.

### Statistical analysis

#### Behaviour

A multi-level Bayesian approach was used to investigate the effects of the experimental manipulations. This allows optimal estimation of population-level effects given the limited participant availability and data imbalances inherent to our clinical setting. Analyses were performed with BRMS^38^ and RStan [Stan Development Team, 2018. RStan: the R interface to Stan. R package version 2.18.2 (http://mc-stan.org/)] running in RStudio v1.3.1056 [RStudio Team, 2020. RStudio: Integrated Development for R. RStudio, Inc., Boston, MA, USA (http://www.rstudio.com/)]. Post-processing and visualization of posterior probabilities was performed in MATLAB v2018a (The MathWorks, Inc., Natick, MA, USA). We examined the experimental effects on three aspects of the saccadic response, each requiring specification of a different response distribution model. Saccadic latency was modelled with a shifted log-normal distribution; binary choice with a logistic model; and latency and choice within a joint drift-diffusion model. Individual model specifications are described below and in the Supplementary material.

##### Latency

To account for skew, response latencies were modelled as a shifted lognormal distribution^39^ with a mean (*μ*) and standard deviation (*σ*) relative to a shift parameter (*δ*, the time of the earliest possible response). The *μ* parameter of the response distribution was allowed to vary with Instruction (Yield, Oppose), Disruption (None, Ipsilateral to fearful face, Contralateral to fearful face) and Emotion (Fearful face, Neutral face) as factors in a fully factorial specification. To absorb potential global lateralization effects,^40^ the Hemifield of fearful face presentation (Left, Right) was modelled as a confound and marginalized out in the subsequent contrasts. The parameters *σ* and *δ* were modelled with log link functions. Subject-level variation was modelled with random intercepts drawn from a hierarchical prior (partial pooling). Four contrasts with specific mechanistic implications were planned.

- A main effect of Disruption presence, capturing task-insensitive effects.
- A main effect of Disruption laterality (contralateral versus ipsilateral to fearful face) restricted to the Yield condition only, capturing the hemifield-specific emotion-dependent effect of disruption on instinctual responses.
- An interaction between disruption laterality (contralateral versus ipsilateral to fearful face) and emotion restricted to the yield condition only, equivalent to the hemifield-specific effect of disruption on instinctual responses invariant to emotional expression.
- An interaction between Instruction, Disruption laterality and Emotion for all conditions, spatially dissociating hemifield-specific effects on detection (sensory) and orientation (motor). Other main effects were also tested for completeness and to ensure behavioural concordance.

##### Choice

The binary choice of orientation target, was modelled with a Bernoulli response distribution with a rate parameter *θ* representing the degree of fearful face preference. *θ* was allowed to vary with Instruction (Yield, Oppose), Disruption (None, Ipsilateral to fearful face, Contralateral to fearful face) and binarized Latency (Early, Late) in a full factorial design. Binarized Latency was derived by performing a median split by latency within each factorial cell (therefore orthogonal to other conditions). Hemifield of fearful face presentation (left, right) was modelled as a confound and marginalized out in the subsequent contrasts. Subject-level variation was modelled with random intercepts drawn from a hierarchical prior (partial pooling). *θ* ranges from 0 to 1 and so was modelled with a logit function. The focus of planned contrasts was clarifying the relation between fear preference and response temporality.

- A main effect of Disruption presence, capturing time-invariant task-insensitive effects.
- A main effect of Disruption laterality (contralateral versus ipsilateral to fearful face) restricted to the Yield condition only, capturing the time-invariant hemifield-specific emotion-dependent effect of disruption on instinctual responses.
- An interaction between Disruption laterality (contralateral versus ipsilateral disruption to fearful face) and Latency restricted to the Yield condition only, capturing the time-dependent hemifield-specific effect of disruption on instinctual responses invariant to emotional expression.
- An interaction between Instruction, Disruption laterality, and Latency (Early, Late) spatially dissociating time-dependent hemifield-specific effects on detection (sensory) and orientation (motor). Other main effects were also tested for completeness and to ensure behavioural concordance.

##### Drift-diffusion

Saccade latency and binary orientation choice were jointly modelled within the influential drift-diffusion model of action selection.^41,42^ This behavioural model views action—here gaze orientation—as the outcome of a single latent competition process noisily accumulating evidence over time until one of two alternative decision thresholds is reached. There are four critical parameters: *a* (the decision threshold) the distance between response boundaries representing response caution; *z* (the bias) the competition starting point capturing prior preference; *v* (the drift-rate) the rate of evidence accumulation towards the outcomes, and *t* (the non-decision time) representing non-decision processes such as sensory delay and motor execution. The drift-rate (*v*) and the bias (*z*) were allowed to vary with Instruction (Yield, Oppose), Disruption (None, Ipsilateral to fearful face, Contralateral to fearful face) and Hemifield of fearful face presentation (Left, Right). The bias, *z*, ranging 0 to 1, was modelled via a logit link function. The two remaining response parameters *a* and *t* were modelled with log link functions and allowed to vary with Instruction (Yield, Oppose) and Disruption (None, Present). We used a mixed-level approach suited to handling data from different participants^41^ in which subject-level variation was modelled separately for each variable with random intercepts drawn from a hierarchical prior (partial pooling). Hemifield was omitted and Disruption modelled with only two levels because these factors are determined before the stimuli appear (the location of the fearful face is not known before each race starts). The contrasts of interest were the main effects and interaction of Instruction (Yield, Oppose) and Disruption (None, Present).

##### Posterior estimation

Weakly-informative priors were applied over model parameters and posterior distributions were estimated using a Hamiltonian Markov Chain Monte Carlo (MCMC) procedure with No-U-Turn sampling (Supplementary material).^43,44^ Posterior contrast estimates of the main effects and interaction effects of interest generated by linear combinations of the posterior parameter chains were summarized by their mean and 95% credibility intervals (CI) (equivalent to the 2.5 and 97.5 percentiles). The probability that a non-zero effect was present in the estimated direction, *P*(effect), was calculated as the posterior probability density either greater than zero (for positive effects) or less than zero (for negative effects). We focused our discussion on the effects with the strongest evidence for which *P*(effect) > 0.95.

#### Face analysis

Pixel-level analysis of the stimuli was conducted to determine which facial morphological features drove orientation. These data were for technical reasons available in only three participants. A pixelwise subtraction of the non-linearly registered versions of each pair of images in the yield condition were subjected to a pixelwise linear fixed-effects model with Disruption (None, Ipsilateral to fearful face, Contralateral to fearful face), and binarized Latency (Early, Late) as factors in SPM12 (http://www.fil.ion.ucl.ac.uk/spm/).^45^ Here, Early versus Late saccades were defined by median split orthogonal to the other conditions. Orientation side (Left, Right) was also modelled as a confound. An omnibus test and pre-planned contrasts were specified to detect features that favoured early rather than late orientation in each of the disruption conditions. Because we were principally interested in the eyes, results were masked to only include the upper half of the face and subsequently thresholded at *P*-value < 0.05 family-wise error (FWE, peak pixel) for inference but are presented at a lower threshold for visualization.

#### Intracranial EEG

##### Preprocessing

The aim of the electrophysiological analysis was to characterize the neural correlates of contralateral face orienting within the amygdala over time. Only data from the Yield undisrupted blocks was analysed because of stimulus artefact during the amygdala disruption block. Intracranial EEG data were analysed using SPM12 and Fieldtrip (http://www.ru.nl/neuroimaging/fieldtrip).^46^ Continuous data were converted to SPM format and re-referenced to a bipolar montage consisting only of the two most distal contacts in the electrode targeted to the amygdala. A 0.1 Hz high-pass filter was applied (two-pass fifth order Butterworth) to remove baseline drift whilst avoiding filter-related phase distortion. With only the bipolar amygdala channel remaining, continuous data were epoched between –2 s and +4 s relative to each trial onset or each saccade. Separate ‘low’ and ‘high’ frequency time-frequency analyses were performed for each trial to allow optimization of smoothing parameters at different frequencies. Low time-frequency analysis was performed between 1 to 48 Hz using a Hanning taper with frequency resolution of 5 Hz, time window of 400 ms and time-steps of 25 ms. For the high frequency analysis, which was performed between 52 Hz and 148 Hz, multitapers using discrete prolate spheroidal sequences were used with a frequency resolution of 10 Hz, time window of 200 ms and time-steps of 25 ms. Time-frequency analyses were subjected to robust-averaging (per condition, per participant), which can remove artefacts without the need to manually specify them. Average 2D time-frequency images were generated per condition per participant and rescaled so that they represented relative percentage increase in power above baseline activity calculated in a window from –600 to –200 ms. Images were smoothed minimally with a 3 × 2 (Frequency × Time) pixel full-width at half-maximum (FWHM) kernel (low frequency images) or 4 × 2 pixel (Frequency × Time) FWHM kernel (high frequency images) to conform to Gaussian error assumptions, prior to statistical modelling.

##### Inference

High frequency and low time-frequency images were masked from 0 to 400 ms relative to trial onset and analysed separately. The analysis was duplicated for epochs triggered to the face stimulus onset and following saccade. For each frequency range, time-frequency data were entered into a 2 × 2 within-subject voxel-wise ANOVA designed to closely reflect the behavioural analysis with factors Recording side (Ipsilateral to fearful face, Contralateral to fearful face) and Emotion (Fearful face, Neutral face). The Hemifield of fearful face presentation (Left, Right) was marginalized out as before. The contrast of interest was an interaction between the two factors which identified electrophysiological correlates of orientation to a contralateral face. Planned *post hoc* two-tailed *F* contrast time-frequency SPMs were thresholded at *P*-value < 0.05 FWE (peak pixel) and are presented overlaid onto equivalent one-tailed *t* contrasts to allow event-related depressions and synchronizations to be distinguished. Marginal beta values and their standard errors were extracted to display the effect-sizes of significant effects of interest.

### Data availability

All code is available from the sources listed above. All behavioural data are available from the authors in anonymized form, subject to a standard material transfer agreement.

## Results

### Unilateral amygdala disruption delays gaze shifts towards contralateral faces

**Figure 2:**
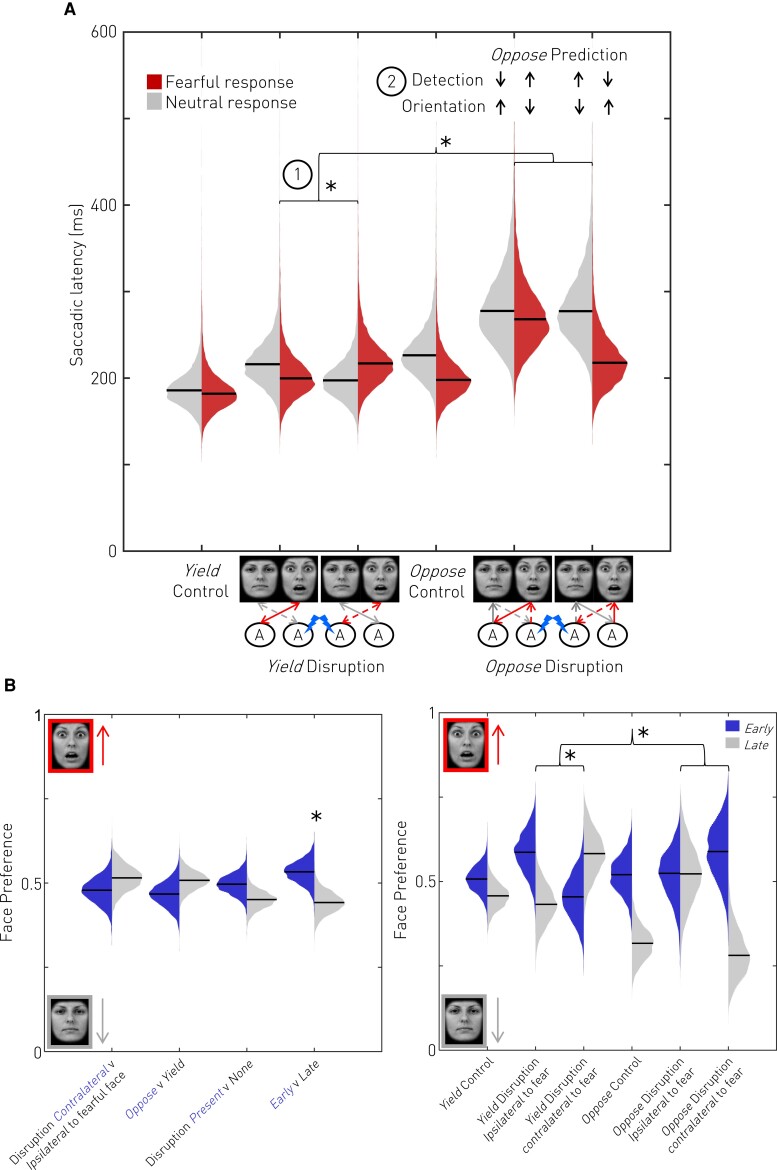
**Effects of unilateral amygdala disruption on task performance.** Posterior mean latency (**A**) and response preference (**B**) are shown as derived from multi-level Bayesian modelling. In the Yield condition unilateral disruption delays contralateral gaze shifts regardless of the emotion of the contralateral face (1). Possible combinations of latencies in the oppose condition, and their interpretation as effects on detection of, or orientation to, faces are depicted as upwards and downwards arrows (2). To distinguish a contralateral detection (sensory) from an automatic orientation (motor) role, participants were asked to perform an Oppose condition, in which the direction of the detected and oriented face were dissociated. If the amygdala mediates rapid contralateral detection, then its disruption should delay ipsilateral Oppose responses, for it then takes longer to detect the target to look away from (2) (see arrows). If the amygdala mediates automatic contralateral orientation, then its disruption should hasten ipsilateral Oppose responses, for it is easier to override the automatic amygdala-mediated orientation to the contralateral field (2) (see arrows). Finally, if the amygdala mediates both detection and orientation, to a degree governed by salience, then a salience-modulated blend of these two effects should be observed. Our results support the third possibility. We observed that amygdala disruption delayed ipsilateral Oppose responses (Interaction of Instruction × Disruption laterality × Emotion) consistent with pure detection, but contralateral Oppose responses varied according to salience of the targeted face: responses away from neutral faces were hastened consistent with detection but responses away from fearful faces were delayed consistent with orientation. In short, the amygdala detects all faces, but preferentially orients towards fearful ones. Disruption conditions on the x-axis are graphically depicted where disruption (blue lightning) of an amygdala (‘A’) is either ipsilateral or contralateral to the presented Fearful face. Arrows from faces towards the amygdala represent theoretical sensory input (always contralateral), whilst arrows from the amygdala towards faces represent subsequent motor response (varies according to Yield and Oppose conditions). Arrows refer to fear (red) or neutral (grey) face processing and are dashed if they are interrupted by disruption. (**B**) Posterior mean face preference as a function of condition. Early and Late are defined by median split. The time-dependent effects of disruption on fear preference mirror the findings from the latency analysis. The earliest responses had a slight preference towards fearful faces (*left*). In the Yield condition, unilateral disruption reduced early preference for the contralateral face regardless of its salience. These preferences were partially inverted by the Oppose condition in a salience-specific manner. Disruption contralateral to a fearful face delayed Oppose responses away from it creating a late preference for the ipsilateral neutral face in line with a detection role. But disruption during presentation of a contralateral neutral face resulted in a stable preference over time: looking away from the contralateral neutral face was delayed because its detection was disrupted but looking away from the ipsilateral fearful face (mediated by the undisrupted amygdala) was also delayed in parallel suggesting a difficulty overriding a drive to automatically orient towards fearful faces.

Bayesian modelling of 1064 Faces task trials (645 non-disrupted, 419 disrupted) demonstrated responses towards a fearful face were lower in latency than those towards a neutral face [main effect of Emotion (Fearful face-Neutral face); Δlatency (95% CI) = –17 (–35 to –2) ms, *P*(effect) = 0.988], revealing that the task engaged neural mechanisms mediating rapid responses towards emotionally salient stimuli. Disruption slowed responses to faces averaged across all conditions [main effect of Disruption presence (Present-None); Δlatency (95% CI) = 32 (19 to 53) ms, *P*(effect) > 0.999]. In the Yield condition, amygdala disruption preferentially slowed contralateral responses regardless of the emotion of the chosen target [interaction of Instruction (Yield only) × Disruption laterality × Emotion; Δlatency (95% CI) = 17 (1 to 37) ms, *P*(effect) = 0.981, Fig. 2A], rather than in an emotion-dependent manner [interaction of Instruction (Yield only) × Disruption laterality; Δlatency (95% CI) = –1 (–17 to 15) ms, *P*(effect) = 0.542, Fig. 2A], establishing the functional lateralization of the human amygdala. This face-specific effect was absent when crosses of varying salience were substituted for faces in the Crosses task (performed in three participants, see the Supplementary material). Note that the failure to demonstrate a dependence on the amygdala of hastened responses to fear does not imply its absence, for its visibility depends on disentangling sensory and motor components, as outlined below.

### Contralateral delay is explained by a blend of detection and fear-specific orientation

To distinguish a sensory role in detecting faces from a motor role in orienting to them, we dissociated the location of the fearful face from the direction of the evoked saccade by asking participants to foveate the face opposite that which captured their attention. In this oppose condition, successful sensory detection in one hemifield generated an orienting motor response in the opposite hemifield, allowing us to compare the impact of disruption lateralized to the motor versus sensory side. We hypothesized that if the amygdala mediates rapid contralateral face detection, then its disruption should delay subsequent ipsilateral oppose responses because it would take longer to detect the target to look away from. If, however, the amygdala mediates automatic contralateral orientation, then its disruption should hasten subsequent ipsilateral oppose responses because it is easier to override the disrupted automatic amygdala-mediated orientation to the contralateral field (see predicted latencies in Fig. 2A). Finally, if the amygdala mediates both detection and orientation, to a degree governed by salience, then a salience-modulated blend of these two effects should be observed.

Our results support this third possibility. We observed that amygdala disruption delayed oppose responses ipsilateral to disruption [interaction of Instruction × Disruption laterality × Emotion; Δlatency (95% CI) = −22 (−46 to −3) ms, *P*(effect) = 0.989, Fig. 2A] as predicted by pure detection, but contralateral oppose responses varied according to the emotion of the targeted face: more hasty away from a neutral face consistent with detection, but relatively delayed away from a fearful face consistent with automatic orientation towards fearful faces. A *post hoc* comparison restricted to the Disrupted oppose conditions confirmed this blend of predictions statistically equivalent to a dependence on Emotion [Instruction (Oppose only) × Disruption (Present only) × Emotion; Δlatency (95% CI) = −42 (−93 to −2) ms, *P*(effect) = 0.980, Fig. 2A]. In short, the amygdala detects all faces, but preferentially orients towards fearful ones.

Further *post hoc* comparisons favoured this account over alternative explanations. In the absence of amygdala disruption, Oppose responses were disproportionately delayed towards neutral faces in comparison with fearful faces relative to their respective Yield baselines [interaction of Instruction × Disruption (None only) × Emotion; Δlatency (95% CI) = −11 (−23 to −1) ms, *P*(effect) = 0.982, Fig. 2A]. This is unlikely to be due to hasty ‘errors’ towards fearful faces because the expected subpopulation of early fearful responses that escape the countermanding process (the ‘errors’) should—and does not—have a mean latency lower than the matching Yield condition (Fig. 2A).^28^ This is also unlikely to be because participants failed to engage with the task, and covertly defaulted to the easier Yield instruction, because the Oppose latencies to fearful and neutral faces are higher than in the Yield condition. The uneven increase in Oppose latencies, therefore, suggests asymmetric difficulty supressing automatic orienting to the detected target: the Oppose instruction reveals a stronger automatic drive to orient towards a more salient (fearful) face.

### Face preference is modulated by amygdala disruption in a time-dependent manner

The effects of amygdala disruption on fear preference were evaluated within a separate Bayesian model of choice. In the absence of disruption, participants chose a fearful face 48% of the time in the Yield condition and 42% of the time in the Oppose condition. There were no clear time-invariant global or hemifield-specific effects of amygdala disruption or interactions of these effects with Instruction (see Supplementary Table 4 for full details). There were, however, time-dependent effects that mirrored the latency analysis. The lowest latency responses had a preference for fearful faces [main effect of Latency; Δrate (95% CI) = 0.11 (0.01 to 0.20), *P*(effect) = 0.988, Fig. 2B] and unilateral amygdala disruption reduced early preference for the contralateral face regardless of its emotion [interaction of Instruction (Yield only) × Disruption laterality × Latency; Δrate (95% CI) = −0.15 (−0.28 to −0.02), *P*(effect) = 0.989, Fig. 2B]. The Oppose instruction inverted this effect of hemifield-specific disruption interaction of Instruction × Disruption laterality × Latency; Δrate (95% CI) = 0.16 (0.04 to 0.27), *P*(effect) = 0.995, Fig. 2B] and again the pattern of oppose responses observed was consistent with a blend of detection and orientation roles of the amygdala. Disruption during presentation of a contralateral fearful face produced a late preference for oppose responses away from it, consistent with detection, but disruption during presentation of a contralateral neutral face resulted in a stable preference over time: looking away from the contralateral neutral face was delayed possibly because its detection was disrupted, but looking away from the ipsilateral fearful face (mediated by the undisrupted amygdala) was also delayed in parallel suggesting a difficulty overriding a drive to automatically orient towards fearful faces.

### The amygdala biases responses towards contralateral fearful faces

**Figure 3:**
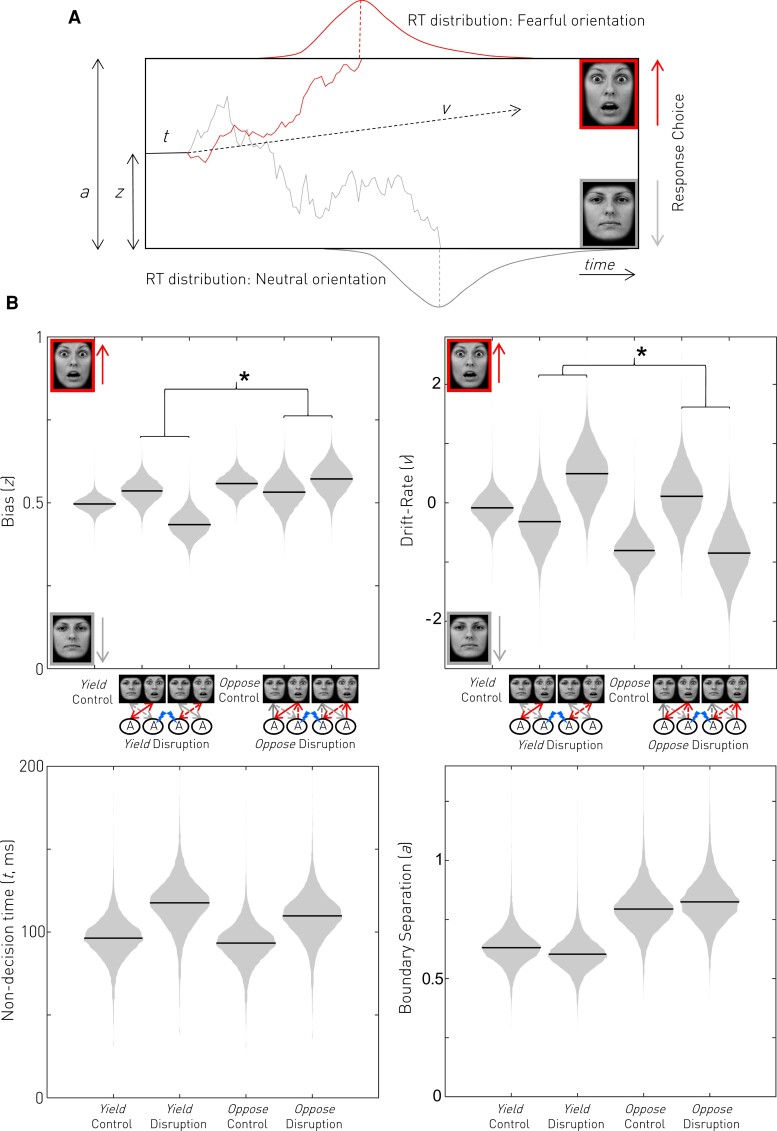
**The drift-diffusion model.** (**A**) Reaction time distributions of a two-choice task can be parameterized in terms of a neural race between two stochastic processes that accumulate evidence towards their respective thresholds. In our task, orientations to fearful (red) and neutral (grey) faces compete. A single instance of each process is shown as an example, leading towards its conditional reaction time distribution. The competition starts at point *z* (the bias) and evidence accumulation is specified in terms of average drift-rate *v*, which can be positive (towards the fearful face) or negative (towards the neutral face). The race terminates when this process reaches threshold *a*. Non-decision confounds are modelled by non-decision time *t*. (**B**) Posterior parameter estimates of the drift-diffusion model suggest unilateral disruption of the amygdala biased responses away from the contralateral face regardless of its salience in the Yield condition. The bias towards the ipsilateral face was altered by the Oppose instruction in a salience-specific manner: bias away from a contralateral fearful face caused by amygdala disruption was reversed by the Oppose instruction: the ipsilateral neutral face remains rapidly detected by the undisrupted amygdala and so Oppose responses away from it are preferred. However, bias away from a contralateral neutral face caused by disruption remains under the Oppose instruction: automatic orientation towards the ipsilateral fearful face mediated by the undisrupted amygdala remains hard to overcome, and so it remains preferred. Drift-rate was affected in the opposite manner suggesting a compensatory mechanism (see main text for explanation). Disruption also increased non-decision time whilst the Oppose instruction increased boundary separation. See Fig. 2 caption for interpretation of graphical *x*-axis labels.

We implemented a Bayesian hierarchical formulation of the influential drift-diffusion model of action selection.^38,41,42^ Changes in the bias parameter of the model (*z*), which has the greatest influence on early responses, were predicted to be most relevant. Unilateral disruption of the amygdala was shown to bias responses away from the contralateral face regardless of its emotion in the Yield condition [interaction of Instruction (Yield only) × Disruption laterality; Δ*z* (95% CI) = −0.104 (−0.196 to −0.009), *P*(effect) = 0.984, Fig. 3B] and the bias towards the ipsilateral face was altered by the Oppose instruction [interaction of Instruction × Disruption laterality; Δ*z* (95% CI) = 0.072 (−0.003 to 0.147), *P*(effect) = 0.984, Fig. 3B] in an emotion-specific manner consistent with the dual detection and orientation role of the amygdala suggested by the independent latency and choice analyses. Specifically, disruption-induced bias away from a contralateral fearful face was reversed by the oppose instruction, rapid detection by the undisrupted amygdala of the ipsilateral neutral face enabling rapid oppose responses away from it. However, disruption-induced bias away from a contralateral neutral face is unaltered under the Oppose instruction: automatic orientation towards the ipsilateral fearful face mediated by the undisrupted amygdala remains hard to overcome.

The effect of amygdala disruption on drift-rate was smaller than, and opposite to the changes in bias in the Yield condition [interaction of Instruction (Yield only) × Disruption laterality; Δ*v* (95% CI) = 1.611 (−0.589 to 3.789), *P*(effect) = 0.926, Fig. 3B]. This effect was also reversed by the Oppose instruction, but without emotion specificity [interaction of Instruction × Disruption laterality; Δ*v* (95% CI) = −3.532 (−6.612 to −0.439), *P*(effect) = 0.987, Fig. 3B]. Disruption increased drift-rate towards the contralateral face in the yield condition and towards the ipsilateral face in the oppose condition, regardless of emotion. This cannot explain the observed latency effects of disruption: drift-rate increases should hasten a response, not delay it as observed. Rather, the drift-rate direction is most consistent with a process that targets the response delayed by amygdala disruption (i.e. compensates for it), and is sensitive to instruction and therefore voluntary control. We propose, therefore, that the drift-rate reflects the activity of non-amygdala-mediated (presumably cortical) compensatory mechanisms. Boundary separation was increased by Instruction [main effect of Instruction; Δ*a* (95% CI) = 0.194 (0.132 to 0.27), *P*(effect) > 0.999, Fig. 3B] consistent with the increased difficulty of the Oppose condition, while amygdala disruption globally increased non-decision time [main effect of Disruption; Δ*t* (95% CI) = 19 (13 to 24) ms, *P*(effect) > 0.999, Fig. 3B]. Full descriptions of parameter estimates are given in the Supplementary material.

### Scleral information drives rapid orienting

**Figure 4:**
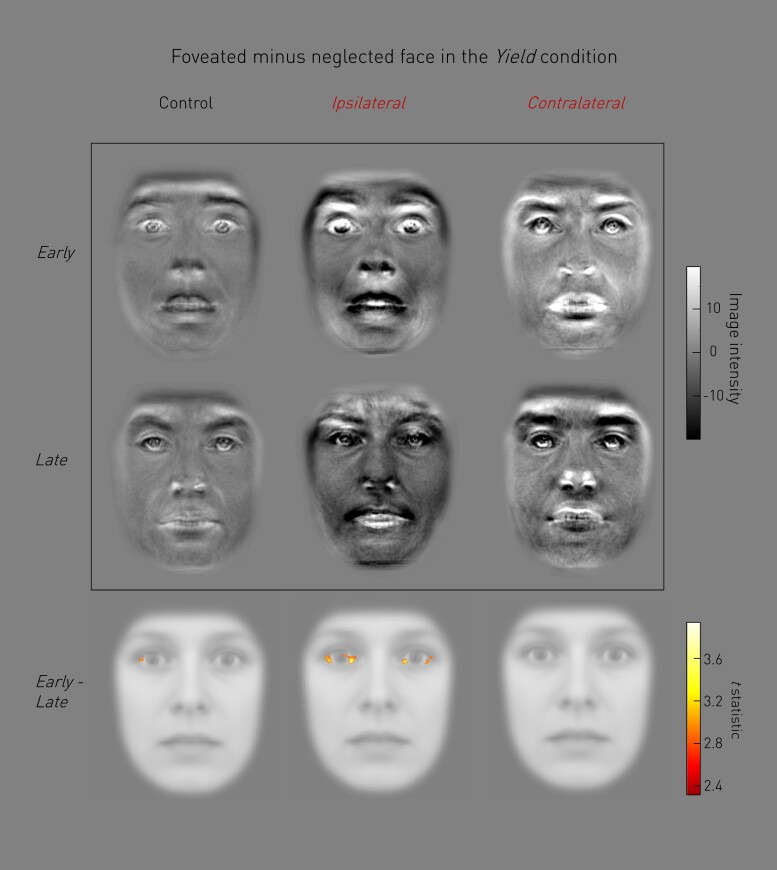
**Amygdala disruption extinguishes early, scleral-driven orientation responses.** Facial feature maps that drove orientation preference were generated trial-by-trial by subtracting the neglected image from the attended image in the Yield condition only. These maps were subjected to a pixelwise linear fixed-effects model with factors Disruption (None, Ipsilateral to fearful face, Contralateral to fearful face) and Latency (Early, Late). Following an omnibus test (Supplementary Fig. 3), *post hoc t-*contrast maps for Early-Late responses are presented for each Disruption condition (*bottom row*) overlaid onto a mean face, with condition means of the raw facial feature maps presented above (*top two rows*). Scleral information significantly drives fast orientation during disruption ipsilateral to a fearful face (*P*-value = 0.017 FWE, where amygdala influence is strongest earlier). There is a non-significant trend in the same direction with no disruption (*P*-value = 0.079 FWE), but this relationship is not detected during disruption contralateral to fearful face. Presented *t-*statistics are thresholded at *P*-value < 0.001 uncorrected for visualization.

It is not the emotion but the facial features in which it is expressed that must direct the choice of visual target. We therefore investigated which facial morphological features, focusing on the upper part of the face, were associated with early versus late responses, regardless of the emotional label. We hypothesized that features known to be accentuated by fear, such as scleral exposure, would be associated with early responding. Scleral information was associated with orientation latency as a function of disruption [omnibus test of Disruption × Latency: peak pixel in left sclera, *F*(3,314) = 7.80, *P*-value = 0.014 FWE; Supplementary Fig. 3]. Planned simple contrasts confirmed that scleral information drove early rather than late orienting during disruption ipsilateral to a fearful face [Disruption (Ipsilateral only) × Latency: peak pixel in right sclera, *t*(1,314) = 3.82, *P*-value = 0.017 FWE, Fig. 4]. A similar, non-significant, trend was seen in the control condition without disruption [Disruption (None only) × Latency: peak pixel in right sclera, *t*(1,314) = 3.33, *P*-value = 0.079 FWE, Fig. 4], but this effect was not detected during disruption contralateral to a fearful face. The contrast in the differential manifestation of expressed fear between the targets of early and late responses—and the effect of stimulation on it—is vividly demonstrated in the pixelwise means of the registered difference images in each condition (Fig. 4).

### Neural oscillations during rapid orientation

**Figure 5:**
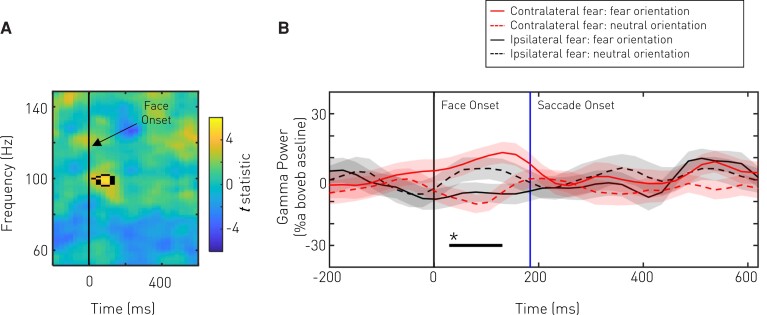
**Amygdala high gamma activity predicts orientation to a contralateral face.** We examined induced amygdala local field potentials during the control (no disruption, Yield only) block to look for correspondence between electrophysiological signals and behaviour. The contrast of interest was an interaction between Recording side (Ipsilateral to fearful face, Contralateral to fearful face) and Emotion (Fearful face, Neutral face). Examination of this interaction aligned to the face stimulus onset (**A**) revealed significant (enclosed by black line) high gamma (99 Hz) activity just after face onset. The timeseries of evolving marginal high gamma induced responses (**B**) suggests that gamma is highest prior to contralateral orientation regardless of face salience. In **B**, activity is represented as mean (solid or dashed line) ± SE (shaded error bars) relative to baseline. Epochs with significant effects are highlighted with a starred horizontal black line.

The electrophysiological signature of the underlying neural processes was revealed in the amygdala local field potentials recorded during the undisrupted Yield blocks of each task. Increased amygdala activity in the high gamma range induced by the face stimulus was found specifically prior to contralaterally oriented gaze shifts regardless of the emotion of the target face [interaction of Recording laterality × Emotion; significant activity between 96–102 Hz at 29–131 ms post face onset; peak voxel at 99 Hz and 106 ms, *F*(1,15) = 27.46, *P*-value = 0.043 FWE (peak level), Fig. 5]. A *post hoc* contrast confined to responses following contralateral fearful face presentation confirmed that gamma activity was similarly increased prior to gaze shifts towards the fearful rather than the neutral face [Recording laterality (Contralateral to fearful face only) × Emotion; significant activity between 95–102 Hz at 55–130 ms post face onset; peak voxel at 98 Hz and 105 ms, *t*(1,15) = 4.82, *P*-value = 0.042 FWE].

Marginal traces of oscillatory activity revealed that high gamma activity increased just after the time of face onset and is a potential analogue of the prior bias to orient contralaterally. No significant effects were found in the lower frequency band or when epochs were triggered to saccade onset.

## Discussion

Drawing on an experimental framework that uniquely enables transient focal disruption of function, here we reconcile conflicting accounts of the role of the amygdala in responding to fear, and provide a putative mechanism for fast, emotional expression-directed orienting. By disentangling distinct components of detection and orientation, and modelling top-down, presumptively cortex-mediated competing effects, we reveal the role of the amygdala to be neither purely sensory nor purely motor, but a context-dependent combination of the two.

The observation of contralateral disruption-induced delay in orienting to any face, regardless of expression, confirms the amygdala’s hemifield specificity. The use of hemifield specific stimuli, and disruption too brief to be compensated by plasticity-dependent adaptation, addresses criticisms levied at discordant observations in humans with unilateral chronic amygdala lesions,^23^ where lateralization effects may have been masked by neural adaptation, obscured by cortical collaterals, or confounded by attentional biases.^40^ Our findings instead cohere with non-human primate anatomy,^16,24^ and with neurophysiological evidence of entrainment to contralateral low-level visual stimuli.^21,22^

We show that a discernible specificity for fear emerges only when the behavioural task dissociates rapidly detecting a contralateral fearful face from automatically orienting towards it. A drift-diffusion framework explains these responses as an amygdala-mediated early bias towards emotionally salient contralateral objects obscured by parallel, slower but potentially more accurate (putatively cortical) detection mechanisms our Oppose manipulation decouples. This reconciles the observation of amygdala-dependent impaired early detection of faces regardless of their emotional valence^17^ with maintenance of specific—even if delayed—fear recognition via parallel pathways.^19^

High-resolution analysis of the facial features driving fast orienting confirms the decisive influence of scleral exposure,^12,23^ narrowing it to fast responses. High gamma activity within the amygdala preceding contralateral orientation suggests anticipatory neural activity^47^ that may bias the subsequent choice of target.

The synoptic picture that emerges from these results is of a fast, spatially-specific amygdala-centred pathway primarily tasked with rapid orienting to manifestations of fear in the immediate environment.

Though inferentially powerful, loss-of-function studies in humans are inevitably constrained by the clinical context. The numbers of participants and stimulation events were naturally restricted on clinical and safety grounds, compelling us to supplement the main task with either the visual salience control task, or the oppose task but not both. A Bayesian approach to analysing the behavioural results enabled us to model optimally both within- and between-subject variance, even in the presence of imbalance across participants. Although generalization from a few or even one patient is constitutionally vulnerable,^10^ the use here of repeated, within-subject, disruption of function, in combination with a highly specific task, and finely parameterized behavioural responses minimizes confounding factors peculiar to the sample. Converging with the aid of tight controls on a sharply defined neural function—the immediate attentional prioritization of signals of threat—further minimizes the impact of collateral, non-specific effects of disruption. More speculatively, phylogenetic conservation of the anatomy is plausibly mirrored by commensurate conservation of function, and consequently variability across the population.

Note that since participants were not specifically instructed to select the fearful face—for our interest is in mechanisms that precede explicit recognition of the emotion—the overall proportion of fearful faces chosen need not be high (48% here in the Yield condition). Varying task instruction and face position have previously been associated with either fear preference^48^ or avoidance^49^ relative to neutral faces^40,50^ and so fearful face position was modelled as a confound in our behavioural analyses. Our participants favoured fearful faces in their lowest latency responses, and the overall preference reduced with the undisrupted Oppose condition (fearful face response 42% of the time) both suggestive of adequate task engagement.

Fundamental neuroscience aside, clarity on the distinctive role of the amygdala is becoming increasingly important clinically as its therapeutic resection—together with closely connected medial temporal structures—becomes more commonly deployed in the treatment of medication-resistant focal epilepsy. That disturbance of affective function is a prominent complication here motivates close attention to the critical role of the mechanisms resection unavoidably disrupts. Moreover, the demonstration of a motor, orienting component to amygdala function is relevant to psychiatric conditions, where the direction of gaze towards others is either reduced (autism)^51^ or increased as part of an orienting bias towards threat in anxious individuals,^52^ and echoes the broader importance of social interaction across the domain.^53^

## Supplementary Material

awac032_Supplementary_Data

## References

[1] Pourtois G , Grandjean D, Sander D, Vuilleumier P. Electrophysiological correlates of rapid spatial orienting towards fearful faces. Cereb Cortex. 2004;14(6):619–633. 15054077 10.1093/cercor/bhh023

[2] Adolphs R . Fear, faces, and the human amygdala. Curr Opin Neurobiol. 2008;18(2):166–172. 18655833 10.1016/j.conb.2008.06.006 PMC2580742

[3] Pessoa L , Adolphs R. Emotion processing and the amygdala: From a “low road” to “many roads” of evaluating biological significance. Nat Rev Neurosci. 2010;11(11):773–782. 20959860 10.1038/nrn2920 PMC3025529

[4] De Gelder B , Van Honk J, Tamietto M. Emotion in the brain: Of low roads, high roads and roads less travelled. Nat Rev Neurosci. 2011;12(7):425. 21673722 10.1038/nrn2920-c1

[5] Pessoa L , Adolphs R. Emotion and the brain: Multiple roads are better than one. Nat Rev Neurosci. 2011;12(7):425.21673722

[6] Morris JS , Frith CD, Perrett DI, et al A differential neural response in the human amygdala to fearful and happy facial expressions. Nature. 1996;383(6603):812–815. 8893004 10.1038/383812a0

[7] Murray RJ , Brosch T, Sander D. The functional profile of the human amygdala in affective processing : Insights from intracranial recordings. Cortex. 2014;60:10–33. 25043736 10.1016/j.cortex.2014.06.010

[8] Hoffman KL , Gothard KM, Schmid MCC, Logothetis NK. Facial-expression and gaze-selective responses in the monkey amygdala. Curr Biol. 2007;17(9):766–772. 17412586 10.1016/j.cub.2007.03.040

[9] Wang S , Yu R, Tyszka JM, et al The human amygdala parametrically encodes the intensity of specific facial emotions and their categorical ambiguity. Nat Commun. 2017;8:14821. 28429707 10.1038/ncomms14821 PMC5413952

[10] Adolphs R , Tranel D, Damasio H, Damasio A. Impaired recognition of emotion in facial expressions following bilateral damage to the human amygdala. Nature. 1994;372(6507):669–672. 7990957 10.1038/372669a0

[11] Adolphs R , Tranel D, Hamann S, et al Recognition of facial emotion in nine individuals with bilateral amygdala damage. Neuropsychologia. 1999;37(10):1111–1117. 10509833 10.1016/s0028-3932(99)00039-1

[12] Adolphs R , Gosselin F, Buchanan TW, Tranel D, Schyns P, Damasio AR. A mechanism for impaired fear recognition after amygdala damage. Nature. 2005;433(7021):68–72. 15635411 10.1038/nature03086

[13] Méndez-Bértolo C , Moratti S, Toledano R, et al A fast pathway for fear in human amygdala. Nat Neurosci. 2016;19(8):1041–1049. 27294508 10.1038/nn.4324

[14] Ledoux JE . The Emotional Brain: The Mysterious Underpinnings of Emotional Life. New York, NY: Simon & Schuster; 1998.

[15] Morris JS , Ohrnan A, Dolan RJ. Conscious and unconscious emotional learning in the human amygdala. Nature. 1998;393(6684):467–470. 9624001 10.1038/30976

[16] Rafal RD , Koller K, Bultitude JH, et al Connectivity between the superior colliculus and the amygdala in humans and macaque monkeys: Virtual dissection with probabilistic DTI tractography. J Neurophysiol. 2015;114(3):1947–1962. 26224780 10.1152/jn.01016.2014 PMC4579293

[17] Gamer M , Schmitz AK, Tittgemeyer M, Schilbach L. The human amygdala drives reflexive orienting towards facial features. Curr Biol. 2013;23(20):R917–R918. 24156808 10.1016/j.cub.2013.09.008

[18] Tsuchiya N , Moradi F, Felsen C, Yamazaki M, Adolphs R. Intact rapid detection of fearful faces in the absence of the amygdala. Nat Neurosci. 2009;12(10):1224–1225. 19718036 10.1038/nn.2380 PMC2756300

[19] Terburg D , Morgan BE, Montoya ER, et al Hypervigilance for fear after basolateral amygdala damage in humans. Transl Psychiatry. 2012;2(5):e115. 22832959 10.1038/tp.2012.46 PMC3365265

[20] Koller K , Hatton CM, Rogers RD, Rafal RD. Stria terminalis microstructure in humans predicts variability in orienting towards threat. Eur J Neurosci. 2019;50(11):3804–3813. 31278789 10.1111/ejn.14504

[21] Peck CJ , Lau B, Salzman CD. The primate amygdala combines information about space and value. Nat Neurosci. 2013;16(3):340–348. 23377126 10.1038/nn.3328 PMC3596258

[22] Peck CJ , Salzman CD. Amygdala neural activity reflects spatial attention towards stimuli promising reward or threatening punishment. Elife. 2014;3:e04478. 25358090 10.7554/eLife.04478 PMC4238057

[23] Gosselin F , Spezio ML, Tranel D, Adolphs R. Asymmetrical use of eye information from faces following unilateral amygdala damage. Soc Cogn Affect Neurosci. 2011;6(3):330–337. 20478833 10.1093/scan/nsq040 PMC3110430

[24] Demeter S , Rosene DL, Van Hoesen GW. Fields of origin and pathways of the interhemispheric commissures in the temporal lobe of macaques. J Comp Neurol. 1990;302(1):29–53. 2086614 10.1002/cne.903020104

[25] Nachev P , Roberts RE, Husain M, Kennard C. The neural basis of meta-volition. Commun Biol. 2019;2(1):1–7. 30886910 10.1038/s42003-019-0346-1 PMC6418118

[26] Leach JCD , Carpenter RHS. Saccadic choice with asynchronous targets: Evidence for independent randomisation. Vision Res. 2001;41:3437–3445. 11718785 10.1016/s0042-6989(01)00059-1

[27] Hanes DP , Carpenter RHS. Countermanding saccades in humans. Vision Res. 1999;39(16):2777–2791. 10492837 10.1016/s0042-6989(99)00011-5

[28] Verbruggen F , Logan GD. Response inhibition in the stop-signal paradigm. Trends Cogn Sci. 2008;12(11):418–424. 18799345 10.1016/j.tics.2008.07.005 PMC2709177

[29] Song J-H , Rafal RD, McPeek RM. Deficits in reach target selection during inactivation of the midbrain superior colliculus. Proc Natl Acad Sci. 2011;108(51):E1433–E1440. 22123965 10.1073/pnas.1109656108 PMC3251126

[30] Amunts K , Kedo O, Kindler M, et al Cytoarchitectonic mapping of the human amygdala, hippocampal region and entorhinal cortex: intersubject variability and probability maps. Anat Embryol (Berl) 2005;210(5-6):343–352. 16208455 10.1007/s00429-005-0025-5

[31] Eickhoff SB , Stephan KE, Mohlberg H, et al A new SPM toolbox for combining probabilistic cytoarchitectonic maps and functional imaging data. Neuroimage 2005;25(4):1325–1335. 15850749 10.1016/j.neuroimage.2004.12.034

[32] Jordan TR , Paterson KB. Re-evaluating split-fovea processing in word recognition: A critical assessment of recent research. Neuropsychologia. 2009;47:2341–2353. 18723038 10.1016/j.neuropsychologia.2008.07.020

[33] Huber A . Homonymous hemianopia after occipital lobectomy. Am J Ophthalmol. 1962;54(4):623–629. 13955389

[34] Mobbs D , Adolphs R, Fanselow MS, et al Viewpoints: Approaches to defining and investigating fear. Nat Neurosci. 2019;22(8):1205–1216. 31332374 10.1038/s41593-019-0456-6 PMC6943931

[35] Ipata AE , Gee AL, Gottlieb J, Bisley JW, Goldberg ME. LIP responses to a popout stimulus are reduced if it is overtly ignored. Nat Neurosci. 2006;9(8):1071–1076. 16819520 10.1038/nn1734 PMC2383314

[36] Jha A , Diehl B, Scott C, McEvoy AW, Nachev P. Reversed procrastination by focal disruption of medial frontal cortex. Curr Biol. 2016;26(21):2893–2898. 27773570 10.1016/j.cub.2016.08.016 PMC5106371

[37] Nowell M , Rodionov R, Diehl B, et al A novel method for implementation of frameless StereoEEG in epilepsy surgery. Oper Neurosurg. 2014;10(4):525–534. 10.1227/NEU.0000000000000544 PMC4224568 25161004

[38] Bürkner PC . brms: An R package for Bayesian multilevel models using Stan. J Stat Softw. 2017;80(1):1–28.

[39] Limpert E , Stahel WA, Abbt M. Log-normal distributions across the Sciences: Keys and Clues: On the charms of statistics, and how mechanical models resembling gambling machines offer a link to a handy way to characterize log-normal distributions, which can provide deeper insight into variability and probability—normal or log-normal: That is the question. Bioscience. 2001;51(5):341–352.

[40] Siman-Tov T , Papo D, Gadoth N, et al Mind your left: Spatial bias in subcortical fear processing. J Cogn Neurosci. 2009;21(9):1782–1789. 18823232 10.1162/jocn.2009.21120

[41] Wiecki TV , Sofer I, Frank MJ. HDDM: Hierarchical Bayesian estimation of the drift-diffusion model in Python. Front Neuroinform. 2013;7:14. 23935581 10.3389/fninf.2013.00014 PMC3731670

[42] Ratcliff R , McKoon G. The diffusion decision model: Theory and data for two-choice decision tasks. Neural Comput. 2008;20(4):873–922. 18085991 10.1162/neco.2008.12-06-420 PMC2474742

[43] Hoffman MD , Gelman A. The no-U-turn sampler: Adaptively setting path lengths in Hamiltonian Monte Carlo. J Mach Learn Res. 2014;15:1593–1623.

[44] Gelman A , Lee D, Guo J. Stan: A probabilistic programming language for Bayesian inference and optimization. J Educ Behav Stat. 2015;40(5):530–543.

[45] Litvak V , Mattout J, Kiebel S, et al EEG and MEG data analysis in SPM8. Comput Intell Neurosci. 2011;2011:852961. 21437221 10.1155/2011/852961 PMC3061292

[46] Oostenveld R , Fries P, Maris E, Schoffelen J-M. FieldTrip: Open source software for advanced analysis of MEG, EEG, and invasive electrophysiological data. Comput Intell Neurosci. 2011;2011:156869. 21253357 10.1155/2011/156869 PMC3021840

[47] Buzsáki G , Wang X-J. Mechanisms of gamma oscillations. Annu Rev Neurosci. 2012;35:203–225. 22443509 10.1146/annurev-neuro-062111-150444 PMC4049541

[48] Lundqvist D , Ohman A. Emotion regulates attention: The relation between facial configurations, facial emotion, and visual attention. Vis Cogn. 2005;12(1):51–84.

[49] Becker MW , Detweiler-Bedell B. Short article: Early detection and avoidance of threatening faces during passive viewing. Q J Exp Psychol. 2009;62(7):1257–1264. 10.1080/17470210902725753 19283559

[50] Hsiao JH , Liu TT. The optimal viewing position in face recognition. J Vis. 2012;12(2):22–22. 10.1167/12.2.22 22375068

[51] Senju A , Johnson MH. Atypical eye contact in autism: Models, mechanisms and development. Neurosci Biobehav Rev. 2009;33(8):1204–1214. 19538990 10.1016/j.neubiorev.2009.06.001

[52] Armstrong T , Olatunji BO. Eye tracking of attention in the affective disorders: A meta-analytic review and synthesis. Clin Psychol Rev. 2012;32(8):704–723. 23059623 10.1016/j.cpr.2012.09.004 PMC3556338

[53] Schilbach L . Towards a second-person neuropsychiatry. Philos Trans R Soc B Biol Sci. 2016;371(1686):20150081. 10.1098/rstb.2015.0081 PMC4685526 26644599

