# Supplementary material for: Orienting to fear under transient focal disruption of the human amygdala
Source: Brain. 2022 Feb 1;146(1):135–48. doi: 10.1093/brain/awac032 (PMC9825557; doi:10.1093/brain/awac032)
Supplement: awac032_Supplementary_Data [file awac032_supplementary_data.pdf]

Supplementary Material

Orienting to fear under transient focal disruption of the human amygdala

ASHWANI JHA[1]*, BEATE DIEHL[1], BRYAN STRANGE[2], ANNA MISEROCCHI[1], FAHMIDA CHOWDHURY[1], ANDREW W MCEVOY[1], PARASHKEV NACHEV[1]*

[1]UCL Queen Square Institute of Neurology, London, UK.

[2]CTB-UPM and Department of Neuroimaging, Reina Sofia Centre for Alzheimer's Research, Madrid, Spain.

A: Supplementary methods

Face Stimuli

Structural Imaging

B: Demographics

Supplementary Table 1. Participant demographics.

C: Bayesian behavioural model specification and estimation

Supplementary Table 2: Bayesian model parametrisation of models of Response Latency, Response Choice and a joint model of both.

Robust Prior Selection

Posterior Estimation

D: The Faces task: additional results for latency and choice

Supplementary Table 3. The effect of amygdala disruption on saccadic latency in the Faces task.

Supplementary Figure 1: Main effects of interest on saccadic latency in the Faces task.

Supplementary Table 4. The effect of amygdala disruption on orientation choice in the Faces task.

E: The Crosses task (low-level visual salience control): methods and results

Supplementary Figure 2: The Crosses Task.

Supplementary Table 5. The effect of amygdala disruption on saccadic latency in the Crosses task.

Supplementary Table 6. The effect of amygdala disruption on orientation choice in the Crosses task.

F: The Faces task: additional results for drift-diffusion model

Supplementary Table 7: Posterior contrast estimates of Bias (*z*) derived from the drift-diffusion model of responses in the Faces task.

Supplementary Table 8: Posterior contrast estimates of Drift-Rate (*v*) derived from the drift-diffusion model of responses in the Faces task.

Supplementary Table 9: Posterior contrast estimates of Boundary Separation (*a*) derived from the drift-diffusion model of responses in the Faces task.

Supplementary Table 10: Posterior contrast estimates of Non-decision time (*t*) derived from the drift-diffusion model of responses in the Faces task.

G: Face analysis: additional results

Supplementary Table 11: Statistical results of the pixelwise face analysis.

Supplementary Figure 3: Early responses are driven by scleral information.

H: Supplementary References

A: Supplementary Methods

Face stimuli

We compiled a set of 89 pairs of images, each pair consisting of a fearful and neutral facial expression posed by the same actor. Images were taken from three databases: the Karolinska Directed Emotional Faces (http://www.emotionlab. se/resources/kdef)[1], the Warsaw Set of Emotional Facial Expression Pictures (http://www.emotional-face.org)[2], and the Radboud Faces Database (http://www.socsci. ru.nl:8180/RaFD2/RaFD)[3]. Each image was processed to minimise variation in spatial and contrast characteristics irrelevant to the expressed emotion. Contrast was linearly normalised in the full 8-bit greyscale range. To align the images, Face++ facial landmark algorithm (https://www.faceplusplus.com) was used to identify 83 facial landmarks per image, from which a rigid transform was computed using coherent point drift registration implemented in Matlab (CPD v2.1[4]). The landmarks were also used to crop each face to the hair and jaw lines, excluding ears, and present it on a black background.

For the pixel-wise analyses, the same images were non-rigidly aligned (to improve pixel-level registration) using a regularised non-linear transform also implemented in CPD. Images were smoothed with a 4 pixel FWHM Gaussian kernel prior to analysis in SPM.

Structural Imaging

Data acquisition

Pre-operatively, whole-brain T1-weighted magnetic resonance imaging of 0.94x0.94x1.1mm resolution was acquired on a 3T General Electric Excite HDx scanner (General Electric, Milwaukee, WI, USA) using an eight-channel array head coil for reception and the body coil for transmission, with standard imaging gradients (maximum strength 40 mT/m and slew rate 150 T/m/s). Post-electrode implantation, the participants underwent an uncontrasted whole-head CT scan of resolution 0.43x0.43x1.2mm. (SOMATOM Definition 128-slice, Siemens Healthcare GmbH, Erlangen, Federal Republic of Germany).

Analysis

We sought to precisely determine the location of the electrode contacts and therefore the electrical disruption sites within the amygdala of each patient in standard stereotactic space. This required two forms of image registration: a native space, within-subject registration of the post-implantation CT to the pre-implantation MRI, and a MNI space, template registration of the MRI, applying the derived parameters so as to secondarily warp the natively registered CT into the same space. We used a previously developed and validated non-linear CT-MRI registration algorithm developed to optimally account for electrode artefact[5]. The procedure is described briefly below.

CT PRE-PROCESSING: a rigid body coregistration to the standard SPM12 tissue probability map was performed based on normalised mutual information with adjustment from a Procrustes analysis weighted by the white and grey matter compartments. This placed the scan in rigid register with the MNI template space.

So as to focus subsequent processing on tissue-relevant contrast, an identical copy of the scan was windowed so as to zero all voxels outwith 0-100 Hounsfield units. This was then filtered with an Oracle-based 3D discrete cosine transform filter[6] to enhance tissue contrast. All subsequent operations were performed on this image, and the final transformation was replicated on the original image at the end.

MR PRE-PROCESSING: As for the CT, a rigid body coregistration to the standard SPM12 tissue probability map was performed based on normalised mutual information with adjustment from a Procrustes analysis weighted by the white and grey matter compartments. This placed the scan in rigid register with the MNI template space, and also with the CT. The scan was then resliced using 4th degree b-spline interpolation to the same bounding box and voxel size as the CT. SPM12's standard unified segmentation and normalisation procedure, with default parameters, was used to generate segmented compartments in native space for each of the standard 6 tissue classes, as well as a set of parameters for non-linear transformation into MNI space of this and any other image in register with it.

NON-LINEAR REGISTRATION OF CT TO MR: This was performed using SPM12's unified segmentation and normalisation procedure on the windowed, filtered CT scan, but instead of using the standard MNI space template tissue compartments as tissue prior probability maps we used the individual MR-derived tissue compartments in native space. The resultant transformation is therefore not into standard stereotactic space but rather the native space of the T1 (which the CT already shares), adjusted to introduce some conformity with the native MR tissue segmentation. Other than removing the affine registration step and any bias correction, the parameters of the algorithm were otherwise as default in SPM12. Note that since SPM12's routine involves explicitly modelling anomalous signal, this adjustment was robust to the artefact created by the metal contacts. The deformation field describing the transformation was applied both to the CT and its corresponding landmark image.

NORMALISATION (TRANSFORMATION INTO MNI SPACE): The deformation field estimated from the MR scan segmentation and normalisation was applied to the grey and white matter compartments of the MR image, transforming them into in standard MNI space. The same deformation field was used to transform the final MR-registered CT into the same space. All transformed images were resliced to 1mm isotropic voxels. The output of MR-derived grey and white matter images, and CT-derived electrode locations were consequently visualised in ParaView (http://www.paraview.org). The location of the amygdala, derived from a 0.1 threshold of the probabilistic maps of amygdala subnuclei derived from the SPM Anatomy Toolbox[7,8] was also superimposed.

B: Demographics

Age	Sex	Handed-ness	Diagnosis	Age of onset of seizures	MRI	Amygdala implanta-tion
38	F	R	Right hemisphere focal epilepsy	13	Small cavernoma right gyrus rectus	R
38	F	L	Left orbitofronal and lateral premotor focal epilepsy	9	Previous left frontal pole resection	L
44	F	L	Right frontal lobe focal epilepsy	14	normal	R
22	M	R	Left temporo-parietal-occipital focal epilepsy	8	normal	L
38	M	Ambidex-trous	Right hemisphere focal epilepsy	4	normal	R
26	F	R	Left cingulate focal epilepsy	14	normal	L

Supplementary Table 1. Participant demographics. All subjects underwent unilateral amygdala implantation with intracranial EEG electrodes as described. L=Left, R=Right.

C: Bayesian behavioural model specification and estimation

Dependent variable (y)	Response distribution (D)	Generalised linear predictor (with link function)	Population-level factors modelled (X)	Priors
**Response Latency**	Shifted lognormal<br>(μ, σ, δ)	$\mu = X\beta_j + Z\eta_s$	INSTRUCTION: DISRUPTION: EMOTION: HEMIFIELD	β_j ~ student_t(5,0,5) *for j=1…J*<br>η_s ~ normal(π, τ) *for s=1…S*<br>π ~ student_t(3,0,2.5)<br>τ ~ halfstudent_t(3,0,2.5)
		$\text{Log}(\sigma) = \alpha + Z\eta_s$	-	α ~ student_t(3,0,2.5)<br>η_s ~ normal(π, τ) *for s=1…S*<br>π ~ student_t(3,0,2.5)<br>τ ~ halfstudent_t(3,0,2.5)
		$\text{Log}(\delta) = \alpha + Z\eta_s$	-	α ~ normal(-3,1)<br>η_s ~ normal(π, τ) *for s=1…S*<br>π ~ student_t(3,0,2.5)<br>τ ~ halfstudent_t(3,0,2.5)
**Response Choice**	Bernoulli(θ)	$\text{Logit}(\theta) = X\beta_j + Z\eta_s$	INSTRUCTION: DISRUPTION: HEMIFIELD	β_j ~ student_t(5,0,5) *for j=1…J*<br>η_s ~ normal(π, τ) *for s=1…S*<br>π ~ student_t(3,0,2.5)<br>τ ~ halfstudent_t(3,0,2.5)
**Response Latency & Choice**	Drift-diffusion<br>(v,z,a,t)	$v = X\beta_j + Z\eta_s$	INSTRUCTION: DISRUPTION: HEMIFIELD	β_j ~ student_t(5,0,5) *for j=1…J*<br>η_s ~ normal(π, τ) *for s=1…S*<br>π ~ student_t(3,0,2.5)<br>τ ~ halfstudent_t(3,0,2.5)
		$\text{Logit}(z) = X\beta_j + Z\eta_s$	INSTRUCTION: DISRUPTION: HEMIFIELD	β_j ~ student_t(5,0,5) *for j=1…J*<br>η_s ~ normal(π, τ) *for s=1…S*<br>π ~ student_t(3,0,2.5)<br>τ ~ halfstudent_t(3,0,2.5)
		$\text{Log}(a) = X\beta_j + Z\eta_s$	INSTRUCTION: DISRUPTION*	β_j ~ student_t(5,0,5) *for j=1…J*<br>η_s ~ normal(π, τ) *for s=1…S*<br>π ~ student_t(3,0,2.5)<br>τ ~ halfstudent_t(3,0,2.5)
		$\text{Log}(t) = X\beta_j + Z\eta_s$	INSTRUCTION: DISRUPTION*	β_j ~ normal(-3,1) *for j=1…J*<br>η_s ~ normal(π, τ) *for s=1…S*<br>π ~ student_t(3,0,2.5)<br>τ ~ halfstudent_t(3,0,2.5)

Supplementary Table 2: Bayesian model parametrisation of models of Response Latency, Response Choice and a joint model of both. For each of the three models, the parameters of the response distribution (D) are given. Response latencies were modelled as a shifted lognormal distribution whose parameters reflect the mean (μ) and standard deviation (σ) of the log-transformed latency, relative to an additional shift parameter (δ, the time of the earliest possible response). The binary choice of orientation target, was modelled with a Bernoulli response distribution with a rate parameter θ representing the degree of Fearful face preference. The drift-diffusion model has four critical parameters: a (the decision threshold) the distance between response boundaries representing response caution; z (the bias) the competition starting point which determines if there

is a prior preference towards one of the outcomes; *v* (the drift-rate) which determines the rate of evidence accumulation towards the outcomes (e.g. easier choices accumulate evidence to one outcome more rapidly) and *t* (the non-decision time). In every model, each parameter is modelled either by an intercept α, or a linear predictor vector β_j of length *J* multiplied by *J* predictors generated from the factorial interaction coding of the population-level factors of interest listed (X). Factorial interactions are specified by a colon (e.g. INSTRUCTION:DISRUPTION specifies the interaction between these two terms) Additionally, all parameters are modelled by vector η_s of length *S* multiplied by *S* subject-specific dummy variables (Z) to account for between-subject variance. Link functions are specified if they were needed to bound parameters to positive numbers (Log) or values between 0 and 1 (Logit). DISRUPTION is modelled as a factor with three levels (*None*, *Ipsilateral* to fearful face, *Contralateral* to fearful face) except where highlighted with a star (*DISRUPTION), in which case it is modelled as a factor with two levels (*Present*, *None*). INSTRUCTION has the levels *Yield* and *Oppose*. EMOTION has the levels *Fearful* face and *Neutral* face. The HEMIFIELD in which the fearful face was presented has the levels *Left* and *Right*. Prior distributions are represented as follows: a normal distribution is represented as normal(mean, standard deviation), a student t distribution is specified as student_t(degrees of freedom, mean, standard deviation) and a positive only student t distribution is specified as halfstudent_t(degrees of freedom, mean, standard deviation).

Robust Prior Selection

We followed a consistent conservative approach employing weakly informative prior distributions over model parameters as described in Supplementary Table 2[9]. Uniform 'improper' priors were avoided as they are implausible and are liable to cause degeneracies in non-linear models. Student t distributions were preferred over Normal distributions were possible owing to their improved behaviour with the heavy tailed data characteristic of behaviour[10]. Priors over continuous unbounded population-level parameters of interest were generally modelled with a Student t distribution (df=5, mean=0, sd=5). Subject-level intercepts were modelled hierarchically with normal priors whose mean and standard deviation were learned from the data (partial pooling) by placing Student t (df=3, mean=0, sd=2.5) and Half-Student t (df=3, mean=0, sd=2.5) priors over the Normal mean and standard deviations respectively. The shift parameter in the latency model and non-decision time in the drift-diffusion model are both linked to, and cannot be higher than, the minimum response latency. We therefore applied tighter constraints to these parameters with a normal prior (mean=-3, sd=1). When transformed this results in a prior predictive distribution with 95% credibility interval between 7-350ms, which covers the reasonable range of values for these parameters.

Posterior Estimation

Calculating the Bayesian posterior for such models can be analytically intractable and therefore posterior parameter estimates were generated using a Markov Chain Monte Carlo (MCMC). BRMS interfaces with RStan (Stan Development Team (2018). RStan: the R interface to Stan. R package version 2.18.2. http://mc-stan.org/) which implements Hamiltonian Monte Carlo with No-U-Turn sampling[11,12]. Relative to the default settings, in all models we increased adapt delta = 0.99 to reduce posterior divergences which often occur in

mixed-level models. Otherwise we used the default recommendations for the Bernoulli and shifted lognormal models in which we ran four chains from random starting values, discarded 2000 warm-up samples per chain (similar but not identical to 'burn-in' in other software) and derived posterior estimates from a further 2000 samples per chain so that the final posterior consisted of 8000 samples in total (no thinning) across all chains. We doubled the number of chains for the drift-diffusion model (4000 discarded warm-up samples and 4000 further posterior samples per chain, 16000 posterior samples in total across all chains) to ensure all chains had an adequate Effective Sample Size (ESS>500 samples). Chain stability and convergence were assessed using visualisation of trace plots, Rhat metrics and ESS, available within the software[12].

D: The Faces task: additional results for latency and choice

Contrast	β (95% CI)	Δlatency (95% CI)	P(effect)
EMOTION (*Fearful* face - *Neutral* face)	-1.238 (-2.353 to -0.156)	-17 (-35 to -2)	0.988*
INSTRUCTION (*Oppose* - *Yield*)	3.471 (2.228 to 4.721)	47 (25 to 80)	>0.999*
DISRUPTION presence (*Present* - *None*)	1.036 (0.7 to 1.379)	32 (19 to 53)	>0.999*
DISRUPTION laterality (*Contralateral* - *Ipsilateral* to fearful face)	-0.683 (-1.764 to 0.398)	-12 (-34 to 7)	0.893
INSTRUCTION[*Yield* only]] x DISRUPTION laterality	-0.03 (-0.558 to 0.494)	-1 (-17 to 15)	0.542
INSTRUCTION[*Yield* only] x DISRUPTION laterality x EMOTION	0.578 (0.033 to 1.122)	17 (1 to 37)	0.981*
INSTRUCTION x DISRUPTION laterality x EMOTION	-1.239 (-2.31 to -0.174)	-22 (-46 to -3)	0.989*
INSTRUCTION x DISRUPTION[*None* only] x EMOTION	-0.198 (-0.387 to -0.013)	-11 (-23 to -1)	0.982*
INSTRUCTION[*Oppose* only] x DISRUPTION[*Present* only] x EMOTION	-0.98 (-1.93 to -0.048)	-42 (-93 to -2)	0.980*

Supplementary Table 3. The effect of amygdala disruption on saccadic latency in the Faces task. Response latencies were modelled as a shifted lognormal distribution. The μ parameter of the response distribution was allowed to vary with INSTRUCTION (*Yield, Oppose*), DISRUPTION (*None*, *Ipsilateral* to fearful face, *Contralateral* to fearful face) and EMOTION (*Fearful* face, *Neutral* face) as factors in a fully factorial specification. HEMIFIELD of fearful face presentation (*Left*, *Right*) was also modelled as a confound and marginalised out in the subsequent contrasts. Four contrasts with specific mechanistic implications were planned. 1) A main effect of DISRUPTION presence, capturing task-insensitive effects. 2) A main effect of DISRUPTION laterality (*Contralateral* vs *Ipsilateral* to fearful face) restricted to the *Yield* condition only, capturing the hemifield-specific emotion-dependent effect of disruption on instinctual responses. 3) An interaction between DISRUPTION laterality (*Contralateral* vs *Ipsilatera*l to fearful face) and EMOTION restricted to the *Yield* condition only, equivalent to the hemifield-specific effect of disruption on instinctual responses invariant to emotional expression. 4) An interaction between INSTRUCTION, DISRUPTION laterality and EMOTION for all conditions, spatially dissociating hemifield-specific effects on detection (sensory) and orientation (motor). Other main effects were also tested for completeness and to ensure behavioural concordance. The posterior mean (and 95% credibility intervals) of contrasts are given as linear combinations of posterior model coefficients β, and also transformed onto the outcome scale (Δlatency) for convenience. The probability of an effect, P(effect), is calculated as the posterior probability density either greater than zero (for positive effects) or less than zero (for negative effects). *Contrasts where P(effect)>0.95 were considered to be important findings. CI = Credibility Interval.

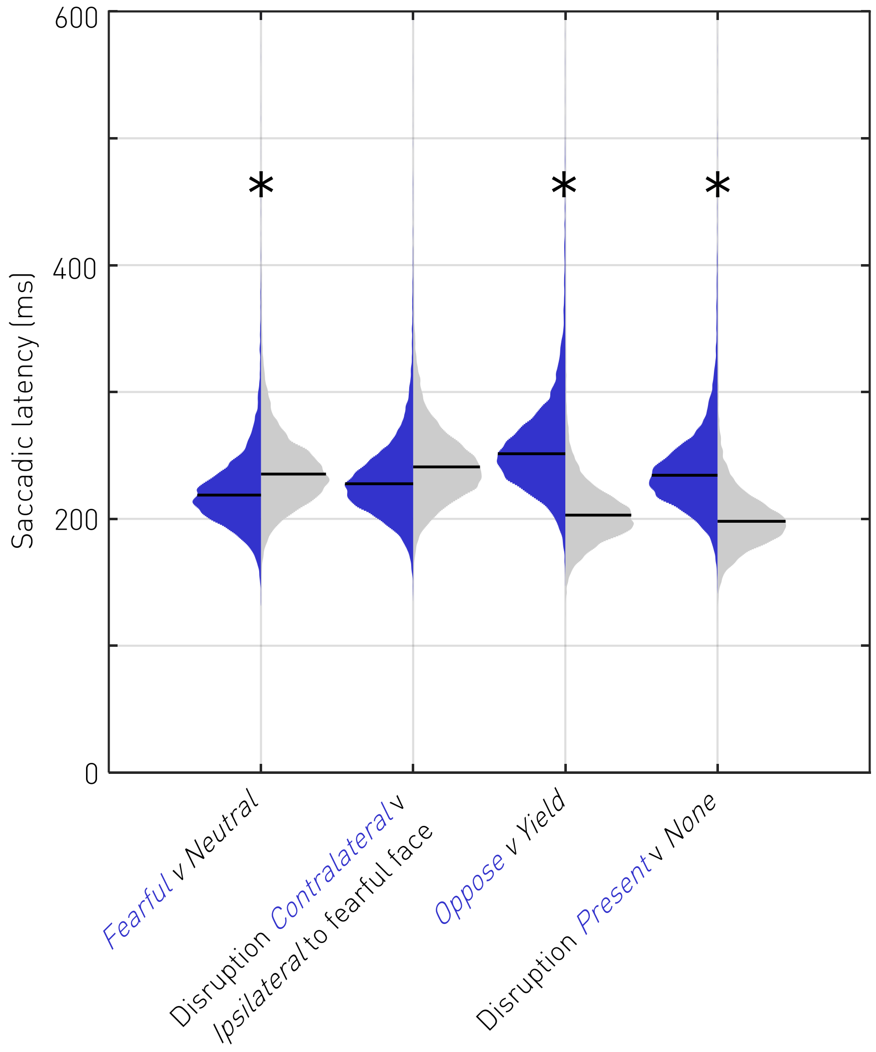

Supplementary Figure 1: Main effects of interest on saccadic latency in the Faces task. Posterior marginal response latencies for the main effects of interest on shifted mean latency ($\mu + \delta$) from the Bayesian shifted-lognormal model are shown. Experimental details are as described in the caption to Supplementary Table 3. For each comparison, the left-hand side distribution refers to the first level of the factor described in the x-axis label - both shown in blue. *Contrasts where P(effect)>0.95 were considered to be important findings.

Contrast	β (95% CI)	Δrate (95% CI)	P(effect)
LATENCY (*Early - Late*)	4.266 (0.519 to 8.119)	0.11 (0.01 to 0.2)	0.988*
INSTRUCTION (*Oppose - Yield*)	-1.886 (-5.927 to 1.953)	-0.05 (-0.15 to 0.05)	0.824
DISRUPTION presence (*Present - None*)	0.737 (-0.449 to 1.912)	0.05 (-0.03 to 0.12)	0.885
DISRUPTION laterality (*Contralateral - Ipsilateral* disruption to fearful Face)	-1.649 (-5.538 to 2.146)	-0.05 (-0.17 to 0.07)	0.800
INSTRUCTION x DISRUPTION presence	0.224 (-0.954 to 1.434)	0.01 (-0.06 to 0.09)	0.645
INSTRUCTION x DISRUPTION laterality	-2.023 (-5.841 to 1.687)	-0.06 (-0.18 to 0.05)	0.856
INSTRUCTION[*Yield* only] x DISRUPTION laterality	0.187 (-2 to 2.413)	0.01 (-0.12 to 0.15)	0.568
INSTRUCTION[*Yield* only] x DISRUPTION laterality x LATENCY	-2.464 (-4.666 to -0.328)	-0.15 (-0.28 to -0.02)	0.989*
INSTRUCTION x DISRUPTION laterality x LATENCY	5.24 (1.43 to 9.007)	0.16 (0.04 to 0.27)	0.995*

Supplementary Table 4. The effect of amygdala disruption on orientation choice in the Faces task. The binary choice of orientation target, was modelled with a Bernoulli response distribution with a rate parameter θ representing the degree of Fearful face preference. θ was allowed to vary with INSTRUCTION (*Yield, Oppose*), DISRUPTION (*None*, *Ipsilateral* to fearful face, *Contralateral* to fearful face) and binarized LATENCY (*Early*, *Late*) in a full factorial design. Binarized Latency was derived by performing a median split by latency within each factorial cell (therefore orthogonal to other conditions). HEMIFIELD of fearful face presentation was modelled as a confound and marginalised out in the subsequent contrasts. Subject was modelled as a random-effect. The focus of planned contrasts was clarifying the relation between fear preference and response temporality. 1) A main effect of DISRUPTION presence, capturing time invariant task-insensitive effects. 2) A main effect of DISRUPTION laterality (*Contralateral* vs *Ipsilateral* disruption to fearful face) restricted to the *Yield* condition only, capturing the time-invariant hemifield-specific emotion-dependent effect of disruption on instinctual responses. 3) An interaction between DISRUPTION laterality (*Contralateral* vs *Ipsilateral* disruption to fearful face) and LATENCY restricted to the *Yield* condition only, capturing the time-dependent hemifield-specific effect of disruption on instinctual responses invariant to emotional expression. 4) An interaction between INSTRUCTION, DISRUPTION laterality, and LATENCY (*Early, Late*) spatially dissociating time-dependent hemifield-specific effects on detection (sensory) and orientation (motor). Other main effects were also tested for completeness and to ensure behavioural concordance. The posterior mean (and 95% credibility intervals) of contrasts are given as linear combinations of posterior model coefficients β, and also transformed onto the outcome scale (Δrate) for convenience. The probability of an effect, P(effect), is calculated as the posterior probability density either greater than zero (for positive effects) or less than zero (for negative effects). *Contrasts where P(effect)>0.95 were considered to be important findings. CI = Credibility Interval.

E: The Crosses Task (low-level visual salience control): methods and results

Supplementary Figure 2

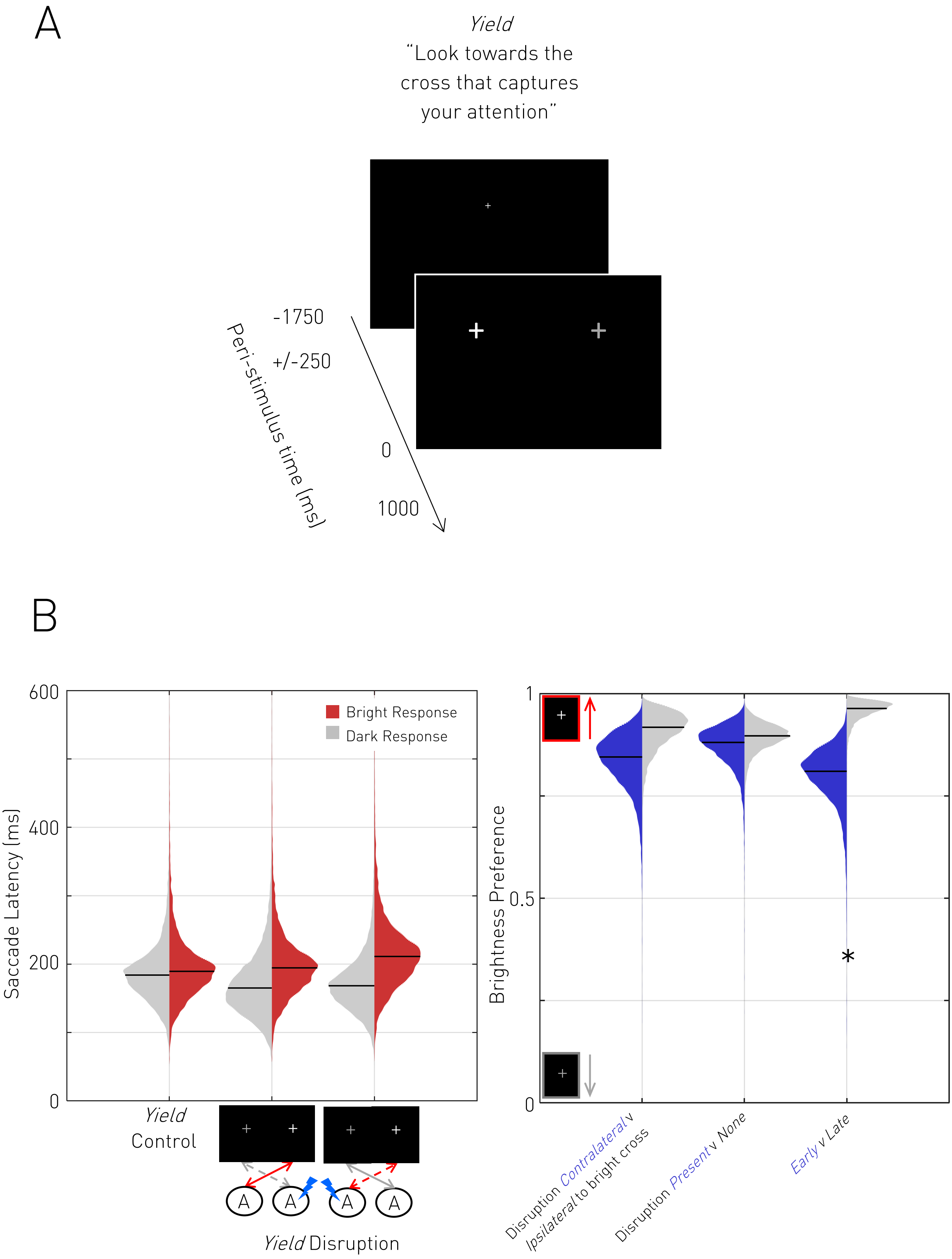

Supplementary Figure 2: The Crosses task. (**A**) In order to determine if the effect of amygdala disruption was specific to faces, we performed a second control variation of the task in which the fearful and neutral facial expressions were replaced by bright and dark crosses leaving all other parameters the same. Only three participants were able to perform the task during amygdala disruption owing to clinical limits on the number of permissible electrical pulses in the amygdala. An *Oppose* condition was also not possible due to this limitation. The data was analysed with a similar strategy to the faces task. **(B):** Response latencies were modelled as a shifted lognormal distribution. The μ parameter of the response distribution was allowed to vary with DISRUPTION (*None*, *Ipsilateral* to bright cross, *Contralateral* to bright cross) and BRIGHTNESS (*Bright* cross, *Dark* cross) as factors in a fully factorial specification. Main effects and interactions of interest were examined and are enumerated in Supplementary Table 5. Critically, the contralateral response delay induced by unilateral amygdala disruption was not seen with the Crosses task (Interaction of DISRUPTION Laterality x BRIGHTNESS; Δlatency [95% CI] = 6 [-29 to 48]ms, P(effect)=0.629, (**B Left**). Indeed, orientation towards *Dark* crosses was *lower* in latency than towards *Bright* crosses (Main effect of Brightness; Δlatency [95% CI] = 26 [1 to 58]ms, P(effect)=0.979) – a pattern opposite to that seen in the **Faces** task. The effects of unilateral amygdala stimulation and salience on latencies seen in the **Crosses** task are, therefore, categorially different to those observed in the **Faces** task. (**C**) The binary choice of orientation target, was modelled with a Bernoulli response distribution with a rate parameter θ representing the degree of preference for a *Bright* cross. θ was allowed to vary with DISRUPTION (*None*, *Ipsilateral* to bright cross, *Contralateral* to bright cross) and binarized LATENCY (*Early, Late*) in a fully factorial design. Binarized LATENCY was derived by performing a median split by latency within each factorial cell (therefore orthogonal to other conditions). Main effects and interactions of interest were examined and are enumerated in Supplementary Table 5. Critically, the reduction in early preference of the contralateral face induced by unilateral amygdala disruption regardless of salience observed in the **Faces** task was *not* seen in the **Crosses** task (Interaction of DISRUPTION laterality x LATENCY; Δrate [95% CI] = -0.034 [-0.183 to 0.088], P(effect) = 0.705, **(B Right)**). Indeed, the most striking effect was that there was an early preference for *Dark* crosses (Main effect of LATENCY; Δrate [95% CI] = -0.154 [-0.305 to -0.059], P(effect)>0.999*, **(B, Right)**). Taken together, these results suggest that the orientation towards *Dark* crosses may be errors (early and less frequent). Although this analysis has reduced power as compared to the **Faces** task due to fewer participants and greater imbalance between salient and nonsalient images (salient images were strongly preferred), there was no similarity between the major findings in the **Faces** task and the low-level **Crosses** tasks. As such, it is likely that mechanistic inferences derived from the Faces task are face-specific, and cannot be generalised to detection of other salient objects. CI = Credibility Interval.

Contrast	β (95% CI)	Δlatency (95% CI)	P(effect)
BRIGHTNESS (*Bright* cross - *Dark* cross)	0.576 (0.025 to 1.132)	26 (1 to 58)	0.979*
DISRUPTION presence (*Present* - *None*)	-0.044 (-0.349 to 0.27)	-3 (-25 to 19)	0.620
DISRUPTION laterality (*Contralateral* - *Ipsilateral* disruption to bright cross)	0.132 (-0.418 to 0.669)	9 (-30 to 47)	0.695
DISRUPTION presence x BRIGHTNESS	0.223 (-0.077 to 0.519)	15 (-5 to 40)	0.932
DISRUPTION laterality x BRIGHTNESS	0.089 (-0.441 to 0.652)	6 (-29 to 48)	0.629

Supplementary Table 5. The effect of amygdala disruption on saccadic latency in the Crosses task. Response latencies were modelled as a shifted lognormal distribution. The μ parameter of the response distribution was allowed to vary with DISRUPTION (*None*, *Ipsilateral* to bright cross, *Contralateral* to bright cross) and BRIGHTNESS (*Bright* Cross, *Dark* Cross) as factors in a fully factorial specification. An *Oppose* condition was not possible due to clinical constraints. Three contrasts with specific mechanistic implications were planned, corresponding to those planned in the Faces task. 1) A main effect of DISRUPTION presence, capturing task-insensitive effects. 2) A main effect of DISRUPTION laterality (*Contralateral* vs *Ipsilateral* to fearful face) restricted to the available *Yield* condition only, capturing the hemifield-specific brightness-dependent effect of disruption on instinctual responses. 3) An interaction between BRIGHTNESS and DISRUPTION laterality (*Contralateral* vs *Ipsilateral* to a bright cross) restricted to the available *Yield* condition only, equivalent to the hemifield-specific effect of disruption on instinctual responses invariant to cross brightness. Other main effects were also tested for completeness and to ensure behavioural concordance. The posterior mean (and 95% credibility intervals) of contrasts are given as linear combinations of posterior model coefficients β, and also transformed onto the outcome scale (Δlatency) for convenience. The probability of an effect, P(effect), is calculated as the posterior probability density either greater than zero (for positive effects) or less than zero (for negative effects). *Contrasts where P(effect)>0.95 were considered to be important findings. CI = Credibility Interval.

Contrast	β (95% CI)	Δrate (95% CI)	P(effect)
LATENCY (*Early* - *Late*)	-5.983 (-10.001 to -2.765)	-0.154 (-0.305 to -0.059)	>0.999*
DISRUPTION presence (*Present* - *None*)	0.09 (-1.929 to 2.309)	-0.001 (-0.093 to 0.071)	0.520
DISRUPTION laterality (*Contralateral* - *Ipsilateral* disruption to bright cross)	-1.051 (-4.537 to 2.506)	-0.041 (-0.193 to 0.083)	0.740
DISRUPTION presence x LATENCY	-0.31 (-2.442 to 1.501)	-0.009 (-0.088 to 0.063)	0.609
DISRUPTION laterality x LATENCY	-0.884 (-4.359 to 2.589)	-0.034 (-0.183 to 0.088)	0.705

Supplementary Table 6. The effect of amygdala disruption on orientation choice in the Crosses task. The binary choice of orientation target, was modelled with a Bernoulli response distribution with a rate parameter θ representing the degree of preference for a *Bright* cross. θ was allowed to vary with DISRUPTION (*None*, *Ipsilateral* to bright cross, *Contralateral* to bright cross) and binarized LATENCY (*Early, Late*) in a fully factorial design. Binarized LATENCY was derived by performing a median split by latency within each factorial cell (therefore orthogonal to other conditions). Results were analysed in a similar fashion to the Faces task bearing in mind that there was no *Oppose* condition. Three contrasts with specific mechanistic implications were planned, corresponding to those planned in the Faces task. 1) A main effect of DISRUPTION presence, capturing time invariant task-insensitive effects. 2) A main effect of DISRUPTION laterality (*Contralateral* vs *Ipsilateral* to fearful face) restricted to the *Yield* condition only, capturing the time-invariant hemifield-specific brightness-dependent effect of disruption on instinctual responses. 3) An interaction between DISRUPTION laterality (*Contralateral* vs *Ipsilateral* disruption to a bright cross) and LATENCY restricted to the available *Yield* condition only, capturing the time-dependent hemifield-specific effect of disruption on instinctual responses invariant to cross brightness. Other main effects were also tested for completeness and to ensure behavioural concordance. The posterior mean (and 95% credibility intervals) of contrasts are given as linear combinations of posterior model coefficients β, and also transformed onto the outcome scale (Δlatency) for convenience. The probability of an effect, P(effect), is calculated as the posterior probability density either greater than zero (for positive effects) or less than zero (for negative effects). *Contrasts where P(effect)>0.95 were considered to be important findings. CI = Credibility Interval.

F: The Faces task: additional results for drift-diffusion model

Contrast	*β* (95% CI)	Δz (95% CI)	P(effect)
INSTRUCTION (*Oppose - Yield*)	1.36 (0.111 to 2.649)	0.068 (0.006 to 0.131)	0.983*
Disruption Presence (*Present - None*)	-0.073 (-0.457 to 0.316)	-0.009 (-0.057 to 0.039)	0.649
Disruption laterality (*Contralateral - Ipsilateral* disruption to fearful face)	-0.507 (-1.753 to 0.744)	-0.032 (-0.109 to 0.046)	0.786
INSTRUCTION x DISRUPTION Presence	0.027 (-0.357 to 0.417)	0.003 (-0.044 to 0.052)	0.557
INSTRUCTION x DISRUPTION laterality	1.166 (-0.049 to 2.385)	0.072 (-0.003 to 0.147)	0.970*
INSTRUCTION[*Yield* only] x DISRUPTION laterality	-0.836 (-1.6 to -0.075)	-0.104 (-0.196 to -0.009)	0.984*
INSTRUCTION[*Oppose* only] x DISRUPTION laterality	0.33 (-0.616 to 1.295)	0.04 (-0.076 to 0.158)	0.750

Supplementary Table 7: Posterior contrast estimates of Bias (*z*) derived from the drift-diffusion model of responses in the Faces task. The bias was allowed to vary with INSTRUCTION (*Yield, Oppose*), DISRUPTION (*None*, *Ipsilateral* to fearful face, *Contralateral* to fearful face) and HEMIFIELD of fearful face presentation (*Left*, *Right*) – the latter marginalised out in subsequent contrasts. The main interaction of interest was between DISRUPTION laterality and INSTRUCTION. Other main effects were also tested for completeness and to ensure behavioural concordance. The posterior mean (and 95% credibility intervals) of contrasts are given as linear combinations of posterior model coefficients β, and also transformed onto the outcome scale (Δz) for convenience. The probability of an effect, P(effect), is calculated as the posterior probability density either greater than zero (for positive effects) or less than zero (for negative effects). *Contrasts where P(effect)>0.95 were considered to be important findings. CI = Credibility Interval.

Contrast	Δv (95% CI)	P(effect)
INSTRUCTION (*Oppose - Yield*)	-2.546 (-5.744 to 0.631)	0.942
DISRUPTION presence (*Present - None*)	0.6 (-0.36 to 1.561)	0.886
DISRUPTION laterality (*Contralateral - Ipsilateral* disruption to fearful face)	-0.31 (-3.399 to 2.766)	0.579
INSTRUCTION x DISRUPTION presence	0.26 (-0.713 to 1.208)	0.703
INSTRUCTION x DISRUPTION laterality	-3.532 (-6.612 to -0.439)	0.987*
INSTRUCTION[*Yield* only] x DISRUPTION laterality	1.611 (-0.589 to 3.789)	0.926
INSTRUCTION[*Oppose* only] x DISRUPTION laterality	-1.921 (-4.093 to 0.227)	0.960*

Supplementary Table 8: Posterior contrast estimates of Drift-Rate (*v*) derived from the drift-diffusion model of responses in the Faces task. The drift-rate was allowed to vary with INSTRUCTION (*Yield, Oppose*), DISRUPTION (*None*, *Ipsilateral* to fearful face, *Contralateral* to fearful face) and HEMIFIELD of fearful face presentation (*Left*, *Right*) – the latter marginalised out in subsequent contrasts. The main interaction of interest was between DISRUPTION laterality and INSTRUCTION. Other main effects were also tested for completeness and to ensure behavioural concordance. The posterior mean (and 95% credibility intervals) of contrasts are given as changes in drift-rate (Δv). The probability of an effect, P(effect), is calculated as the posterior probability density either greater than zero (for positive effects) or less than zero (for negative effects). *Contrasts where P(effect)>0.95 were considered to be important findings. CI = Credibility Interval.

Contrast	β (95% CI)	Δa (95% CI)	P(effect)
INSTRUCTION (*Oppose - Yield*)	0.545 (0.408 to 0.676)	0.194 (0.132 to 0.27)	>0.999*
DISRUPTION presence (*Present - None*)	-0.006 (-0.126 to 0.112)	-0.002 (-0.045 to 0.04)	0.54
INSTRUCTION x DISRUPTION presence	0.081 (-0.037 to 0.199)	0.029 (-0.013 to 0.073)	0.911

Supplementary Table 9: Posterior contrast estimates of Boundary Separation (*a*) derived from the drift-diffusion model of responses in the Faces task. The boundary separation was allowed to vary with INSTRUCTION (*Yield, Oppose*) and DISRUPTION (*None, Present*). The contrasts of interest here were the main effects and interaction of these two factors. The posterior mean (and 95% credibility intervals) of contrasts are given as linear combinations of posterior model coefficients β, and also transformed onto the outcome scale (Δa) for convenience. The probability of an effect, P(effect), is calculated as the posterior probability density either greater than zero (for positive effects) or less than zero (for negative effects). *Contrasts where P(effect)>0.95 were considered to be important findings. CI = Credibility Interval.

Contrast	β (95% CI)	Δt (95% CI)	P(effect)
INSTRUCTION (*Oppose - Yield*)	-0.101 (-0.148 to -0.051)	-0.005 (-0.008 to -0.002)	>0.999*
DISRUPTION presence (*Present - None*)	0.36 (0.312 to 0.405)	0.019 (0.013 to 0.024)	>0.999*
INSTRUCTION x DISRUPTION presence	-0.038 (-0.081 to 0.011)	-0.002 (-0.004 to 0.001)	0.94

Supplementary Table 10: Posterior contrast estimates of Non-decision time (*t*) derived from the drift-diffusion model of responses in the Faces task. The non-decision time was allowed to vary with INSTRUCTION (*Yield, Oppose*) and DISRUPTION (*None, Present*). The contrasts of interest here were the main effects and interaction of these two factors. The posterior mean (and 95% credibility intervals) of contrasts are given as linear combinations of posterior model coefficients β, and also transformed onto the outcome scale (Δt) for convenience. The probability of an effect, P(effect), is calculated as the posterior probability density either greater than zero (for positive effects) or less than zero (for negative effects). *Contrasts where P(effect)>0.95 were considered to be important findings. CI = Credibility Interval.

G: Face analysis: additional results

Contrast	Peak pixel	statistic	*p* (FWE)
DISRUPTION x LATENCY (*Early* – *Late*) (*None*, *Ipsilateral* to fearful face, *Contralateral* to fearful face)	Right eye<br>Left eye	$F(3,314)=7.31$<br>$F(3,314)=7.80$	0.024*<br>0.014*
DISRUPTION[*None* only] x LATENCY	Right Eye	$t(1,314)=3.33$	0.079
DISRUPTION[*Ipsilateral* only] x LATENCY	Right Eye<br>Left Eye	$t(1,314)=3.82$<br>$t(1,314)=3.63$	0.017*<br>0.032*

Supplementary Table 11: Statistical results of the pixelwise Face analysis. Pixel-level analysis of the stimuli was conducted to determine which facial morphological features drove orientation. These data were for technical reasons available in only three participants. A pixelwise subtraction of the non-linearly registered versions of each pair of images in the *Yield* condition were subjected to a pixelwise linear fixed-effects model with DISRUPTION (*None, Ipsilateral* to fearful face, *Contralateral* to fearful face), and binarized LATENCY (*Early, Late*) as factors. ORIENTATION side (*Left*, *Right*) was also modelled as a confound. An omnibus test and pre-planned contrasts were specified to detect features that favoured early, rather than late orientation in each of the disruption conditions (interactions between DISRUPTION x LATENCY). Because we were principally interested in the eyes, results were masked to only include the upper half of the face and subsequently thresholded at $p<0.05$ FWE (peak pixel) for inference. The peak pixel in all clusters surviving $p<0.001$ uncorrected are also presented above for completeness.

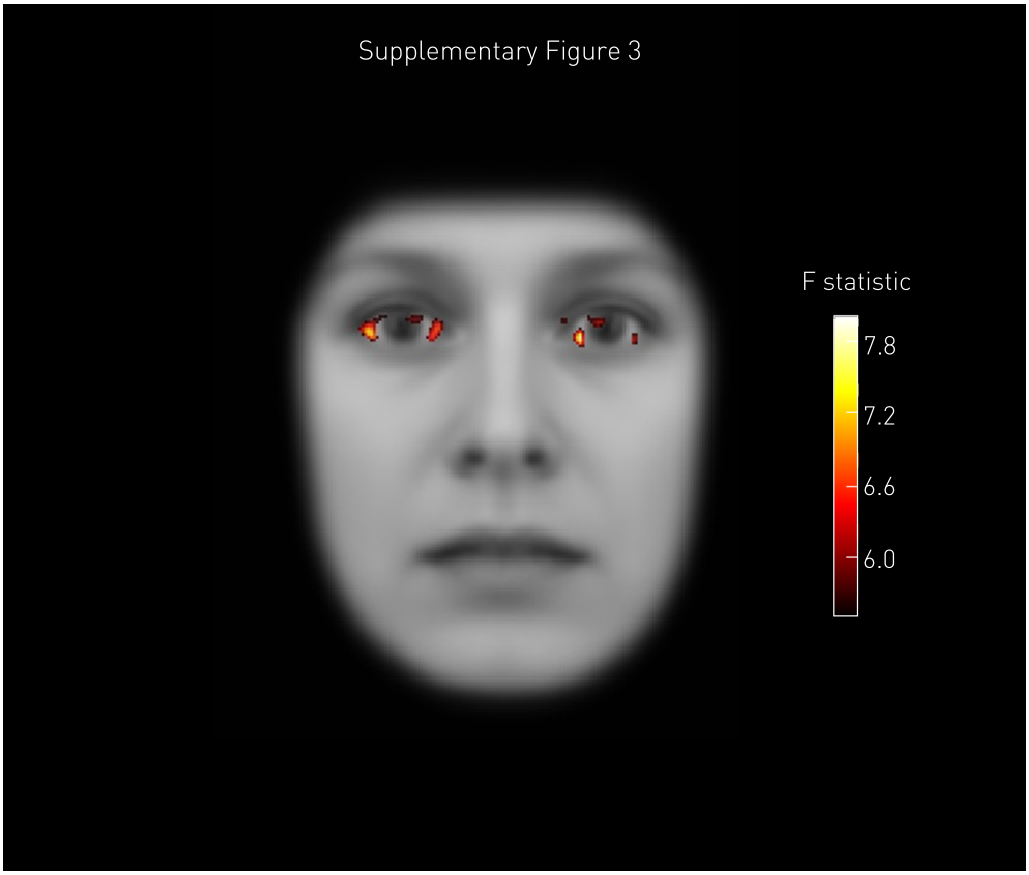

Supplementary Figure 3: Early responses are driven by scleral information. Pixel-level analysis of the stimuli was conducted to determine which facial morphological features drove orientation. An Omnibus test of DISRUPTION x LATENCY revealed that scleral information was associated with orientation latency was associated with orientation latency as a function of disruption (see Supplementary Table 11). The *F*-statistic map for this contrast is displayed above overlaid onto a mean face. Presented *F*-statistics are thresholded at $p<0.001$ uncorrected for visualisation. *Post hoc t*-tests within each disruption condition are presented in Figure 4.

H: Supplementary References

1. Lundqvist D, Flykt A, Öhman A. *The Karolinska Directed Emotional Faces– KDEF*. Department of Clinical Neuroscience, Psychology Section, Karolinska Institutet; 1998.

2. Olszanowski M, Pochwatko G, Kuklinski K, Scibor-Rylski M, Lewinski P, Ohme RK. Warsaw set of emotional facial expression pictures: a validation study of facial display photographs. *Front Psychol*. 2014;5:1516. doi:10.3389/fpsyg.2014.01516

3. Langner O, Dotsch R, Bijlstra G, Wigboldus DHJ, Hawk ST, van Knippenberg A. Presentation and validation of the Radboud Faces Database. *Cogn Emot*. 2010;24(8):1377-1388. doi:10.1080/02699930903485076

4. Myronenko A, Song X, Carreira-Perp MA. Non-rigid point set registration: Coherent Point Drift. *Proc Adv Neural Inf Process Syst*. 2007:1009-1016,.

5. Jha A, Diehl B, Scott C, McEvoy AW, Nachev P. Reversed Procrastination by Focal Disruption of Medial Frontal Cortex. *Curr Biol*. 2016;26(21). doi:10.1016/j.cub.2016.08.016

6. Manjón J, Coupé P, Buades A, Collins DL, Robles M. New methods for MRI denoising based on sparseness and self-similarity. *Med Image Anal*. 2012;16(1):18-27. doi:10.1016/j.media.2011.04.003ï

7. Amunts K, Kedo O, Kindler M, et al. Cytoarchitectonic mapping of the human amygdala, hippocampal region and entorhinal cortex: intersubject variability and probability maps. *Anat Embryol (Berl)*. 2005;210(5-6):343-352. doi:10.1007/s00429-005-0025-5

8. Eickhoff SB, Stephan KE, Mohlberg H, et al. A new SPM toolbox for combining probabilistic cytoarchitectonic maps and functional imaging data. *Neuroimage*. 2005;25(4):1325-1335. doi:10.1016/j.neuroimage.2004.12.034

9. Gelman A, Jakulin A, Pittau MG, Su YS. A weakly informative default prior distribution for logistic and other regression models. *Ann Appl Stat*. 2008;2(4):1360-1383. doi:10.1214/08-AOAS191

10. O'Hagan A. On Outlier Rejection Phenomena in Bayes Inference. *J R Stat Soc Ser B*. 1979;41(3):358-367. doi:10.1111/j.2517-6161.1979.tb01090.x

11. Hoffman MD, Gelman A. The no-U-turn sampler: Adaptively setting path lengths in Hamiltonian Monte Carlo. *J Mach Learn Res*. 2014;15:1593-1623.

12. Gelman A, Lee D, Guo J. Stan: A Probabilistic Programming Language for Bayesian Inference and Optimization. *J Educ Behav Stat*. 2015;40(5):530-543. doi:10.3102/1076998615606113
